# Unveiling how intramolecular stacking modes of covalently linked dimers dictate photoswitching properties

**DOI:** 10.1038/s41467-019-13428-3

**Published:** 2019-12-02

**Authors:** Ru-Qiang Lu, Xiao-Yun Yan, Lei Zhu, Lin-Lin Yang, Hang Qu, Xin-Chang Wang, Ming Luo, Yu Wang, Rui Chen, Xiao-Ye Wang, Yu Lan, Jian Pei, Wengui Weng, Haiping Xia, Xiao-Yu Cao

**Affiliations:** 1 State Key Laboratory of Physical Chemistry of Solid Surfaces, Collaborative Innovation Center of Chemistry for Energy Materials (iChEM), Key Laboratory for Chemical Biology of Fujian Province, Department of Chemistry, College of Chemistry and Chemical Engineering, Xiamen University, 361005 Xiamen, China; 2 Department of Polymer Science, College of Polymer Science and Polymer Engineering, The University of Akron, Akron, OH 44325-3909 USA; 3 School of Chemistry and Chemical Engineering, Chongqing University, 400030 Chongqing, China; 4 State Key Laboratory of Elemento-Organic Chemistry, College of Chemistry, Nankai University, 300071 Tianjin, China; 5 Beijing National Laboratory for Molecular Sciences (BNLMS), the Key Laboratory of Bioorganic Chemistry and Molecular Engineering of Ministry of Education, College of Chemistry and Molecular Engineering, Peking University, 100871 Beijing, China

**Keywords:** Optical materials, Organic chemistry, Photochemistry

## Abstract

Covalently linked π-stacked dimers represent the most significant platform for elucidating the relationship between molecular alignments and their properties. Here, we present the one-pot synthesis of two intramolecularly π-stacked dimers and disclose how intramolecular stacking modes dictate photoswitching properties. The dimer, which features cofacially stacked chromophores and geometrically favours intramolecular photochemical [2 + 2] cycloadditions, displays a nearly irreversible photoswitching behaviour. By contrast, the dimer, bearing crosswise stacked chromophores, is geometrically unfavourable for the cycloaddition and exhibits a highly reversible photoswitching process, in which the homolysis and reformation of carbon−carbon single bonds are involved. Moreover, the chiral carbon centres of both dimers endow these photoswitches with chirality and the separated enantiomers exhibit tuneable chiroptical properties by photoswitching. This work reveals that intramolecular stacking modes significantly influence the photochemical properties of π-stacked dimers and offers a design strategy toward chiral photoswitchable materials.

## Abstract (web summary)

Covalently bridged π-stacked dimers are excellent molecular platforms for understanding the relationship between stacking orientation and properties. Here, the authors synthesize a pair of π-stacked dimers that are aligned either cofacially or crosswise, allowing them to compare how the intramolecular stacking mode affects each dimer’s photoswitching properties.

## Introduction

Since the first discovery and subsequent synthesis of [2.2]paracyclophane in the middle of the 20th century^1,2^, covalently linked π-stacked dimers have attracted intense research interest, ranging from developing new building blocks and synthetic strategies to investigating chemical consequences of their geometries and through-space electronic couplings between π-conjugated moieties^3–6^. Among these research focuses, the chemical reactivity arising from molecular geometries is of significant interest to generate novel functional properties. For example, benefited from the well-defined alignments of the two π-moieties, geometrically preferred reactions, such as photo-induced cycloadditions, can be achieved efficiently^7–9^. Another important character of these dimers is their high intramolecular steric repulsion, which may lead to unusually long carbon–carbon single bonds (bond length greater than 1.6 Å)^10–12^. The elongated C−C single bonds are accompanied by the decreasing of bond dissociation energies^13^ and thus the increasing of reactivity, for instance, via thermal- or photo-activated bond cleavage^14,15^. These remarkable structural features and their accompanying potential photochemical reactivity make covalently linked dimers promising candidates for photo-responsive materials. Nevertheless, the on-demand control over the relative positions of the neighbouring π-moieties of such dimers, although highly important for modulating the properties, remains a challenge.

Photoswitchable molecules, which interconvert reversibly between two or more isomers through light irradiation or heat, have received intense attention owing to their wide applications in electronics^16–19^, photopharmacology^20,21^, and molecular machines^22,23^. Transferring the chirality into a photoswitch enables the reversible and precise control over chiral information. Many unconventional applications for photoswitches, such as in manipulating supramolecular chirality^24,25^, liquid crystalline properties^26–30^, and asymmetric catalysis^31^, therefore can be achieved. For instance, Li et al. successfully employed chiral photoswitches as dopants to alter the helical axis of cholesteric liquid crystals^27^. Common photoswitchable building blocks such as azobenzene, diarylethene, and spiropyran, however, do not show chirality or cannot maintain their chirality upon photoswitching. The introduction of stable chirality to them usually requires the additional chiral substituent(s)^27,32–35^ or the chiral environment^36^.

Herein, we report a one-pot synthesis of cyclopentane-bridged dimeric molecules (**3a** and **3b**) through cascade nucleophilic additions. The intramolecular stacking modes and thus photoswitching properties can be effectively modulated by changing the substituents on the aromatic skeleton. The well-aligned alkene pairs in *endo* dimer (**3a**) easily leads to intramolecular photo-induced [2 + 2] cycloaddition, which is nearly irreversible upon UV irradiation at a shorter wavelength. In contrast, the *exo* dimer (**3b**) displays outstanding reversible photoswitching properties. The photoisomerization of **3b**, undergoing through biradical intermediates, shows excellent fatigue resistance under ambient conditions and large changes in geometries and dipole moments after photoswitching. To our knowledge, incorporating the dimers with different intramolecular stacking modes into photoswitches has never been reported. Furthermore, the chiroptical properties of each enantiomer of **3b** can be dramatically modulated by UV irradiation. The enantiopure **3b** represents a novel kind of photoswitchable building block bearing chiral characteristics, which is a significant complement to the well-established photoswitches. This work, from the fundamental point of view, provides a deep understanding of the relationship between stacking modes in covalently linked dimers and their photoswitching properties. These results would be important in the future design of photoswitches.

## Results

### Synthesis of covalently linked dimers

**Fig. 1:**
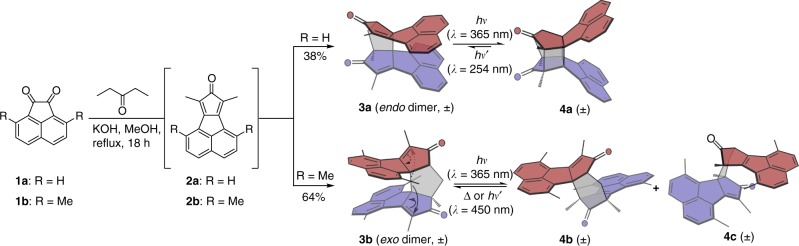
One-pot synthesis and photochemical reactions of **3a** and **3b**. Both **3a** and **3b** are obtained as a pair of enantiomers. The photochemical rearrangements of **3b** leading to **4b** and **4c** are indicated by solid and dashed arrows, respectively.

The synthesis of dimers **3a** and **3b** was inspired by a serendipitous finding during the preparation of cyclopentadienone **2b** (Fig. 1), a key synthetic intermediate of corannulene^37–39^. Aldol condensation of 4,7-dimethylacenaphthenequinone (**1b**) and 3-pentanone produces compound **2b** in the presence of KOH in methanol after refluxing for 1 h^40^. An unprecedented dimer **3b** based on cyclopentadienone, however, was obtained in 64% yield when the reaction time was elongated to 18 h. Different from the previous work in Diels–Alder (D–A) dimerization of cyclopentadienone^41^, five-membered-ring-bridged dimers are formed in this study due to the presence of KOH, a strong base, in the reaction mixture. To further investigate this reaction, we replaced **1b** (R = Me) with acenaphthenequinone (**1a**, R = H) as the starting material. Instead of forming an *exo* dimer like **3b**, an *endo*-dimer **3a** was obtained in 38% yield. The photoisomerization of **3a** and **3b** is also displayed in Fig. 1, which will be discussed in details later. All new compounds were characterized by nuclear magnetic resonance (NMR) and high-resolution mass spectrometry (HRMS).

### Single-crystal analysis of the dimers

**Fig. 2:**
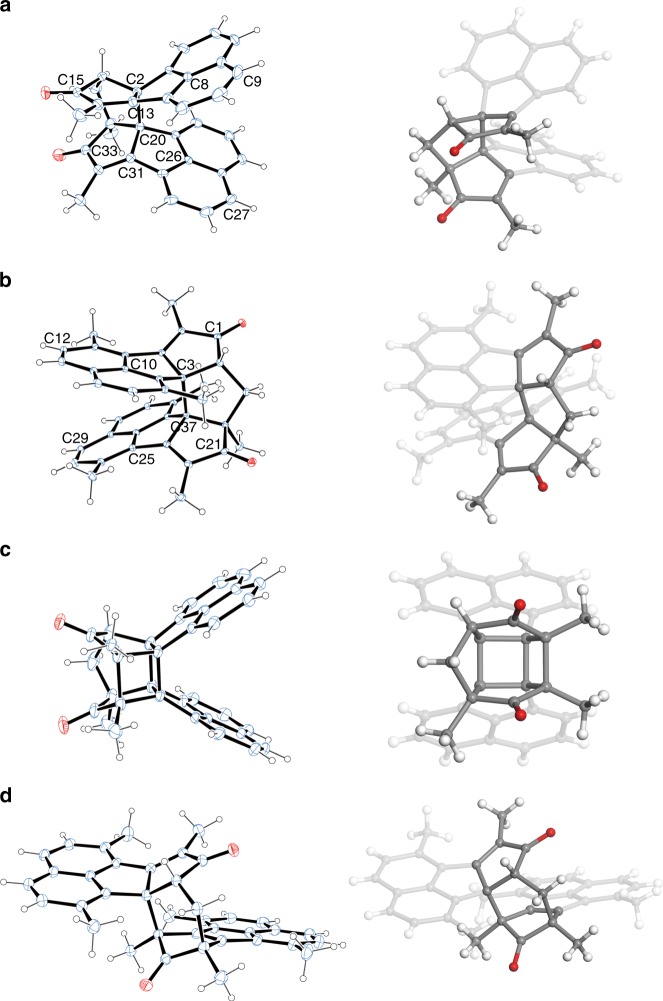
Single-crystal structures. **a** **3a**, **b** **3b**, **c** **4a**, and **d** **4b**. Thermal displacement ellipsoids are shown at the 25% probability level. The annulated rings are highlighted.

The structures and configurations of **3a** and **3b** are unambiguously confirmed by single-crystal X-ray analysis (Fig. 2 and Supplementary Table 1). The single-crystal structures of **4a** and **4b** are also shown here, which will be discussed later. A pair of enantiomers are observed for both **3a** and **3b** in single crystals (Supplementary Fig. 1). In dimers **3a** and **3b**, two conjugated monomers are bridged by a five-membered ring. In dimer **3a**, the monomers are aligned on the same side of the bridge, hence giving an *endo* configuration. From the top view of **3a**, two chromophores containing alkene moieties are nearly cofacially aligned. The stacking angle (*θ*), as defined in Supplementary Table 2, was determined as 11.4°. The distances between two intramolecular stacked arms are ranging from 2.67 to 5.29 Å (Supplementary Table 2). Arising from the steric hindrance between two chromophores, the length of the bond connecting two chromophores (C2–C20 single bond) is 1.66 Å, much larger than the typical length for a carbon–carbon single bond (1.54 Å)^42^. Dimer **3b** exhibits an *exo* configuration with the two chromophores stacked crosswise. The stacking angle (*θ*) is determined as 102.8°, much larger than that of **3a** (Fig. 2b). The distances between face-to-face benzenes are ranging from 3.31 to 4.45 Å (Supplementary Table 2). The C3–C37 single bond exhibits a length of 1.62 Å, which is also larger than the typical length of carbon–carbon single bond.

### Mechanism of dimerization

**Fig. 3:**
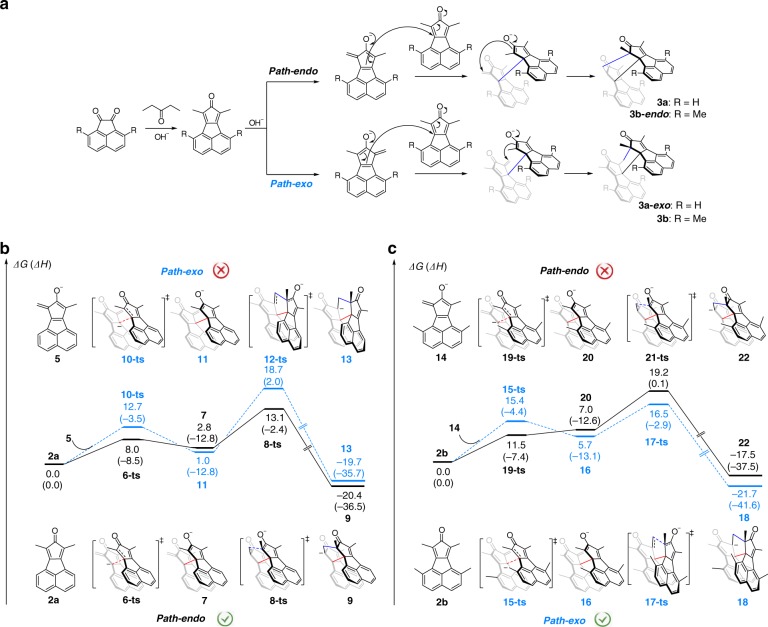
Proposed mechanism. **a** Proposed mechanism of the nucleophilic addition cascade process. Free energy profiles of *Path-endo* (black) and *Path-exo* (blue) for the dimerization of **b** reactant **2a** and **c** reactant **2b**. Values (in kcal·mol^−1^) represent the relative free energies calculated by the ωB97X-D//M11 method in methanol. The values in parentheses are the relative enthalpy.

To give insights into the reaction mechanism of the dimerization process, a cascade process of nucleophilic additions is proposed (Fig. 3). The mechanism for the formation of **3a** is illustrated in Fig. 3a (**3b** was formed in a similar process but with a different stereoselectivity). The proposed mechanism can be roughly divided into three stages. The first stage is an Aldol condensation between **1a** and 3-pentanone, producing the dehydrated product **2a**. This reaction has been thoroughly investigated, and **2a** can be separated out^40^. The second stage is a nucleophilic addition between two cyclopentadienonyl derivatives (**2a**) after one of them is deprotonated on the *β*-carbon of its carbonyl group. In this step, two out of four chiral centres are determined, and the linkage between monomers is formed, producing enolates **7**. In the third step, a Michael addition takes place, thus presenting the final configuration.

To elucidate the origin of stereoselectivities during the formation of **3a** and **3b**, density functional theory (DFT) calculations were employed. Taking **3a** for example, two competitive pathways leading to **3a** or **3a**-***exo*** are considered and the free energy profiles are summarized in Fig. 3b and Supplementary Table 3. The total free energy of cyclopentadienonyl **2a** and its conjugate base **5** (which could be generated by deprotonation of **2a** in the presence of base) is set as the relative zero in the free energy profiles. In the *Path-endo*, the initial intermolecular nucleophilic addition of **5** to the benzyl position on **2a** via transition state **6-ts** generates enolate complex **7** with a free energy barrier of 8.0 kcal·mol^−1^. The subsequent intramolecular cyclization leads to the formation of intermediate **9**, from which the *endo* dimeric product **3a** can be generated after protonation. The overall activation free energy of *Path-endo* was calculated as 13.1 kcal·mol^−1^. Alternatively, the isomer **3a-*exo*** can also be generated in a similar way (*Path-exo*). The corresponding activation free energy of this process (via **12-ts**) was calculated to be 18.7 kcal·mol^−1^, much higher than that of *Path-endo* (via **8-ts**), indicating that the generation of **3a** in the *endo* configuration is more favourable. Non-covalent interaction (NCI) analysis^43^ reveals that the energetic discrepancy is mainly contributed by intramolecular π–π interactions between two fragments lowering the energy of transition state **8-ts** (Supplementary Fig. 2). However, when methyl groups are introduced on naphthalene in the case of **3b**, the steric hindrance of methyl groups results in a high activation free energy of 19.2 kcal·mol^−1^ in *Path-endo*, which is higher than that in *Path-exo* (16.5 kcal·mol^−1^, Fig. 3c), leading to the preferable formation of **3b** in the *exo* configuration. These theoretical calculations confirm that both π-π interactions and steric hindrance play important roles in the stereoselectivity of this reaction and illustrate that how the small changes of substituents (H vs. Me) afford completely different configurations.

### Photoswitching properties of the dimers

Initial insights into the photochemical behaviours of cyclopentadione dimers produced by D–A reactions in 1980s^41,44,45^ inspired us to investigate the photoswitching properties of the two obtained dimers. The photoisomerization results of **3a** and **3b** is shown in Fig. 1. The parallel alkene pairs in **3a** geometrically favour the photochemical [2 + 2] cycloaddition to produce **4a**. The intramolecular cycloaddition reaction of **3a** in CHCl_3_ was monitored by in-situ UV-vis absorption spectroscopy, which indicates that the reaction finishes in less than 16 s upon UV irradiation at 365 nm (Supplementary Fig. 3). The quantitative conversion from **3a** to **4a** was demonstrated by in-situ ^1^H NMR spectroscopy (Supplementary Fig. 4). The cycloaddition reaction can even happen in single crystals, which was confirmed by in-situ single-crystal X-ray diffraction of **3a** before and after UV irradiation. The cycloaddition is irreversible upon heating at 50 °C and nearly irreversible upon UV irradiation at a shorter wavelength (*λ* = 254 nm). Upon UV irradiation at 254 nm for 9.5 h, less than 4% of **4a** undergoes photocleavage of the cyclobutane to recover to **3a** (Supplementary Fig. 4). Nonetheless, the four chiral centres on **3a** endow its enantiopure forms, i.e. RSSS-**3a** and SRRR-**3a** (Supplementary Fig. 1), with tuneable chiroptical properties upon UV irradiation (Supplementary Fig. 12a and Fig. 12b).

**Fig. 4:**
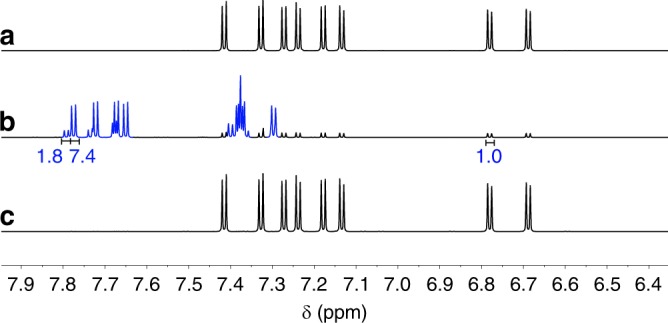
Photo- and thermal-isomerization monitored by NMR. ^1^H NMR spectra of **3b** **a** in the pristine state, **b** in the photostationary state ([**4b** + **4c**]/[**3b**] = 90/10 from the integrations of the peaks at 7.77−7.80 ppm and 6.78−6.79 ppm) and **c** in the recovered state after being heated at 40 °C for 8 h in CD_2_Cl_2_ (4.6 mM).

**Fig. 5:**
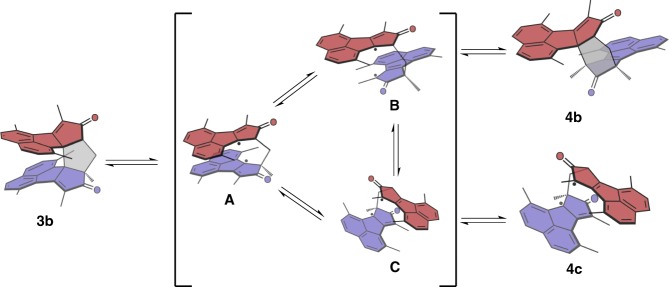
Proposed biradical mechanism of the photoswitching process of **3b**. The photoisomerization from **3b** to **4b** was shown in Supplementary Movie 1.

Dimer **3b**, however, exhibits completely different photoisomerization behaviours. Two new sets of signals appeared in ^1^H NMR upon UV irradiation at 365 nm (Fig. 4b). This photoisomerization is reversible upon heating. After being heated at 40 °C for 8 h, the mixture completely changed to the initial state (Fig. 4c). The photoisomerization products of **3b** can be separated out from the starting material by column chromatography over silica gel. A yellow solid containing two compounds (i.e. **4b** and **4c**, which cannot be separated due to their similar polarities and thus are characterized as a mixture) was obtained. The [**4b**]:[**4c**] ratio is around 4.1: 1 (determined from the integrations of the peaks at 7.77–7.78 ppm for **4b** and at 7.79–7.80 ppm for **4c** in ^1^H NMR spectra). The structure of the main portion **4b** was determined by single-crystal X-ray analysis (Fig. 2d). One of the arms of **4b** isomerizes to an acenaphthylene derivative and its relative orientation to the other arm is also changed. σ[l,3] shifts were first considered as the possible mechanism of the isomerization, but it should be noted that the thermal [1,3] shifts proceeding through suprafacial shifts are forbidden^46^. We therefore proposed a biradical mechanism to explain this isomerization (Fig. 5). Upon UV irradiation, the overlong C3−C37 single bond (Fig. 2), resulting from the steric repulsion between two cyclopentadienone derivatives, is broken to form biradical intermediate **A**, which has two resonant structures **B** and **C**. The biradicals of **B** and **C** form new carbon−carbon single bonds to produce **4b** and **4c**, respectively. The ^1^H NMR spectrum of the less portion is in accordance with the structure of **4c** (Supplementary Fig. 5), which confirms the proposed mechanism. This homolytic bond breaking and formation mechanism is also found in imidazole-based photoswitches^47,48^. DFT calculations reveal that the energy of **4b** is about 1 kcal·mol^−1^ (ωB97X-D/6-31 G(d,p) in the gas phase) lower than that of **4c**, which accounts for the larger population of **4b** than that of **4c** in the photostationary state (PSS). The conversion percentage from **3b** to **4b** and **4c** in the PSS determined by high-performance liquid chromatography (HPLC) is 93% (Supplementary Fig. 6), in agreement with the ^1^H NMR result (90%). The similar isomerization via biradical intermediates was not observed for **3a**, although the length of C2−C20 bond for **3a** is 1.66 Å, probably because the rate of photo-induced cycloaddition is much faster than that of the isomerization through biradical intermediates.

This photoisomerization results in large difference in geometries and dipole moments between **3b** and its photoisomers. Compounds **4b** and **4c** show similar dipole moments, geometries, and absorption (Supplementary Table 4 and Supplementary Fig. 7). Therefore, even though they cannot be easily separated, they can still be regarded as one portion when they are used as a photoswitch. Herein, the main portion **4b** is used as an example to illustrate the difference from **3b**. The end-to-end distances are increased from 4.7 Å for **3b** to 8.8 Å for **4b** (Supplementary Table 4), generating a difference of 4.1 Å in length, which is larger than that of the *E/Z* isomerization of azobenzene (~3.5 Å^17^). This large geometrical change in chemical structures is expected to induce significant morphological difference in hierarchical assemblies and thus provides the possibilities in the control of protein folding and unfolding^49^, artificial molecular muscles^50^ and supramolecular chemistry^51^. In addition, the dipole moments change from 6.2 Debye for **3b** to 2.7 Debye for **4b** due to the shift of relative orientations of two arms. To reveal kinetic processes of the thermal isomerization from **4b** and **4c** to **3b**, time-dependent ^1^H NMR spectra of the solution of **3b** in the PSS at various temperatures were measured. The thermal isomerizations of **4b** and **4c** follow first-order kinetics (Supplementary Fig. 8 and Supplementary Table 5). At room temperature (298 K), the activation energy of the thermal isomerization from **4b** to **3b** was determined to be 24.8 kcal·mol^−1^ with a half-life of 3.87 h and that from **4c** to **3b** was 24.5 kcal·mol^−1^ with a half-life of 4.05 h.

**Fig. 6:**
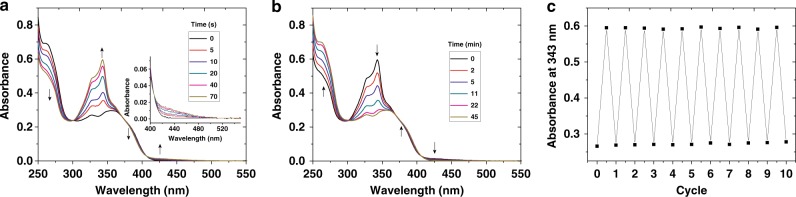
Photo- and thermal-isomerization monitored by absorption spectra. Absorption of **3b** **a** upon UV irradiation 365 nm and **b** then heated at 55 °C (CHCl_3_, 2.24 × 10^-5^ M); **c** absorbance at 343 nm upon UV irradiation at 365 nm for 70 s and then heated at 55 °C for 45 min in repeated cycles.

The photoswitching process of **3b** was also monitored by UV-vis spectroscopy (Fig. 6a-c). Three isobestic points at 300 nm, 371 nm and 406 nm are observed upon UV irradiation at 365 nm. The absorbance from 300 nm to 369 nm and from 406 nm to 500 nm increases as the UV irradiation time extends. The onset absorbance red-shifts from 428 to 500 nm. The photoisomerizations reach the PSS in less than 70 s. After being heated at 55 °C for less than 45 min, the mixture recovers to pristine state entirely. To test the photostability of **3b**, **4b**, and **4c**, the solution of **3b** was alternately irradiated with UV light and heated, and the absorbance at 343 nm was selected to monitor the photoswitching process. The switch exhibits no fatigue after 10 repeated cycles under ambient conditions (Fig. 6c, temperature: ~28 °C, humidity: 75–90%), indicating a large fatigue resistance of the photoswitching process. To confirm if the thermal-back isomerization can be achieved by light irradiation, the PSS solution of **3b** was irradiated with visible light at 450 nm. The back isomerization from **4b** and **4c** to **3b** is indeed accelerated (Supplementary Fig. 9), revealing the photoisomerization of **3b** is reversible both upon heating and light irradiation.

**Fig. 7:**
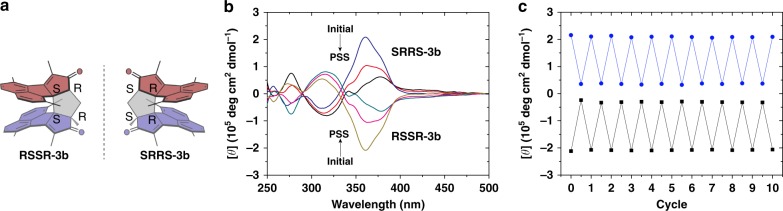
Chiral photoswitching of **3b**. **a** Enantiomers of **3b**. **b** CD spectra of RSSR-**3b** and SRRS-**3b** upon UV irradiation at 365 nm. **c** Molar ellipticity at 360 nm upon UV irradiation at 365 nm for 70 s and then heated at 55 °C for 45 min in repeated cycles (SRRS-**3b** in blue line and RSSR-**3b** in black line).

The enantiomers of **3b** (RSSR-**3b** and SRRS-**3b**) can be separated by chiral HPLC (Supplementary Fig. 10b). Comparison of their circular dichroism (CD) spectra and the corresponding DFT-simulated ones reveals that the component eluting at 13.6 min is RSSR-**3b**, and that eluting at 15.7 min is RSSR-**3b** (Supplementary Fig. 11b). As expected, RSSR-**3b** and SRRS-**3b** show mirror-like CD spectra (Fig. 7b). The chiroptical properties of RSSR-**3b** and SRRS-**3b** can be altered by UV irradiation at 365 nm. For example, the exciton couplet of SRRS**-3b** at 360 nm weakens as the irradiation time increases and fully recovers after being heated at 55 °C for 45 min (Supplementary Figs. 12c and 12d). The molar ellipticity of SRRS**-3b** at 360 nm is switched from 2.1 × 10^5^ (ON state) to 3.7 × 10^4^ deg·cm^2^·dmol^-1^ (OFF state) with a ON/OFF ratio at 5.7 upon UV irradiation. The chiral properties exhibit no decay after ten repeated switching cycles (Fig. 7c). Such high fatigue resistance under ambient conditions outperforms that of most reported chiral photoswitches^32,35,47^. These outstanding properties make the chiral photoswitches highly promising building blocks for the applications in modulating supramolecular chirality, chiral information storage, asymmetric synthesis, etc.

## Discussion

In summary, a new type of cyclopentane-bridged dimeric molecules with different intramolecular stacking modes have been synthesized in a concise one-pot procedure. Their intramolecular stacking modes (i.e. *endo* or *exo* configurations) can be modulated by modifying the substituents on the aromatic core, as confirmed by single-crystal structures. Theoretical calculations reveal that such stereoselectivity resulted from the competition between steric repulsion and π–π interactions. The *endo*-dimer (**3a**) displays a nearly irreversible photoswitching process through intramolecular photochemical [2 + 2] cycloaddition and its reverse reaction. The *exo*-dimer (**3b**) undergoes reversible photoswitching processes via biradical intermediates. This work demonstrates the possibility of tuning intramolecular stacking modes of rigidly linked π-stacked molecules by altering the substituents and provides a chiral photoswitchable building block, i.e. *exo*-dimer, with excellent properties such as high fatigue resistance and large changes in geometries, chiroptical properties, and dipole moments during photoisomerizations. The *exo*-dimer is a significant complement to the well-established photoswitches as exemplified by azobenzene, diarylethene, and spiropyran. Further fine-tuning of the photoswitching properties and exploration of their potential applications, for example, in single-molecule electronics^52,53^, singlet fissions^54,55^, bioimaging^56^, and supramolecular chemistry^49,51,57,58^, require the facile chemical modifications of the *exo*-dimer. The introduction of reactive groups such as halogens within the precursors may provide photoswitching dimers allowing for versatile late-stage functionalization, which is in progress in our group.

## Methods

### Synthesis of 3a

A solution of KOH (862 mg, 15.4 mmol) in methanol (10 mL) was added dropwise to a mixture of **1a** (350 mg, 1.92 mmol), 3-pentanone (661 mg, 7.68 mmol) and methanol (10 mL) at room temperature. The mixture was stirred at reflux for 18 h. The precipitation was filtered, washed with water and dried under vacuum. The crude product was further purified by column chromatography over neutral alumina (eluent: chloroform/hexane = 3/7) to give **3a** (168 mg, 38 %) as a pale-yellow solid.

### Synthesis of 3b

Compound **3b** was synthesized using the same methods as described for **3a**. It was obtained as a pale-yellow solid (277 mg, 64%).

The detailed procedures and characterization are presented in Supplementary Methods. All NMR spectra are shown in Supplementary Figs. 13–29.

### Single-crystal X-ray diffraction

Single-crystal diffraction data were collected on a XtaLAB Synergy, Dualflex, HyPix single-crystal diffractometer using Cu Kα (*λ* = 1.54184 Å) micro-focus sealed X-ray tube at 100 K for **3a**, **3b** and **4a** and on a Rigaku SuperNova X-Ray single-crystal diffractometer using Cu Kα (*λ* = 1.54184 Å) micro-focus X-ray sources at 150 K for **4b**.

### Computational methods

DFT calculations on mechanisms were carried out at the ωB97X-D/6-311 + G (d,p)//M11/6-31 G(d) levels of theory in methanol using a SMD solvation model. DFT calculations on geometries and TD-DFT calculations on optical spectra were performed at the ωB97X-D/6-31 G(d,p) level of theory in the gas phase. The coordinates of all optimized structures are provided in Supplementary Data 1. Detailed methods are presented in the Supplementary Methods.

### Kinetic measurements

The solution of **3b** in CDCl_3_ (3.85 mM) was irradiated with UV light (*λ* = 365 nm) to the photostationary state and used immediately. The solution of the mixture of **3b**, **4b**, and **4c** was kept at various temperature (303, 308, 313, 318, and 323 K) in dark and characterized by time-dependent ^1^H NMR spectra. The temperature was controlled by a water bath. The ambient temperature was around 25 °C. The samples were dipped in ice water for 15 s before the acquisition of ^1^H NMR spectra to reduce the decay of the photoisomers resulting from the lingering warmth during the measurements. The concentrations of **3b**, **4b**, and **4c** in the mixture were determined by the integrations of the peaks with the chemical shifts at 1.19 ppm for **3b**, 0.87 ppm for **4b**, and 0.77–0.78 ppm for **4c**. The initial concentrations were around 2.85 mM for **4b** and around 0.61 mM for **4c**.

## Supplementary information

Supplementary Information

Description of Additional Supplementary Files

Supplementary Data 1

Supplementary Movie 1

## Data Availability

All data used for this paper are available from the authors on request. The X-ray crystallographic coordinates for structures reported in this study have been deposited at The Cambridge Crystallographic Data Centre (CCDC) under deposition numbers 1913518 for **3a**, 1939142 for **3b**, 1832043 for **4a**, and 1832044 for **4b**. These data can be obtained free of charge from The Cambridge Crystallographic Data Centre at www.ccdc.cam.ac.uk/data_request/cif.

## References

[1] Brown CJ, Farthing AC (1949). Preparation and structure of di-*p*-xylylene. Nature.

[2] Cram DJ, Steinberg H (1951). Macro Rings. I. Preparation and spectra of the paracyclophanes. J. Am. Chem. Soc..

[3] Ghasemabadi PG, Yao T, Bodwell GJ (2015). Cyclophanes containing large polycyclic aromatic hydrocarbons. Chem. Soc. Rev..

[4] Liu Z, Nalluri SKM, Stoddart JF (2017). Surveying macrocyclic chemistry: from flexible crown ethers to rigid cyclophanes. Chem. Soc. Rev..

[5] Canevet D, Pérez EM, Martín N (2011). Wraparound hosts for fullerenes: tailored macrocycles and cages. Angew. Chem. Int. Ed..

[6] Boydston AJ (2001). [2.2]Paracyclophane/dehydrobenzoannulene hybrids: transannular delocalization in open-circuited conjugated macrocycles. Angew. Chem., Int. Ed..

[7] Yoshizawa M, Tamura M, Fujita M (2006). Diels-Alder in aqueous molecular hosts: unusual regioselectivity and efficient catalysis. Science.

[8] Hayashi T (1976). Excimer fluorescence and photodimerization of anthracenophanes and 1,2-dianthrylethanes. J. Am. Chem. Soc..

[9] Brogaard RY (2011). Pseudo-bimolecular [2+2] cycloaddition studied by time-resolved photoelectron spectroscopy. Chem. Eur. J..

[10] Suzuki T, Takeda T, Kawai H, Fujiwara K (2008). Ultralong C-C bonds in hexaphenylethane derivatives. Pure Appl. Chem..

[11] Dodziuk, H. *Strained Hydrocarbons* (Wiley-VCH, 2009).

[12] Ishigaki Y, Shimajiri T, Takeda T, Katoono R, Suzuki T (2018). Longest C–C single bond among neutral hydrocarbons with a bond length beyond 1.8 Å. Chem.

[13] Zavitsas AA (2003). The relation between bond lengths and dissociation energies of carbon−carbon bonds. J. Phys. Chem. A.

[14] Rüchardt C, Beckhaus HD (1980). Towards an understanding of the carbon-carbon bond. Angew. Chem. Int. Ed..

[15] Koch TH, Olesen JA, DeNiro J (1975). Unusually weak carbon-carbon single bond. J. Am. Chem. Soc..

[16] Jia C (2016). Covalently bonded single-molecule junctions with stable and reversible photoswitched conductivity. Science.

[17] Russew M-M, Hecht S (2010). Photoswitches: from molecules to materials. Adv. Mater..

[18] Zhang J, Zou Q, Tian H (2013). Photochromic materials: more than meets the eye. Adv. Mater..

[19] Wang L, Li Q (2018). Photochromism into nanosystems: towards lighting up the future nanoworld. Chem. Soc. Rev..

[20] Velema WA, Szymanski W, Feringa BL (2014). Photopharmacology: beyond proof of principle. J. Am. Chem. Soc..

[21] Broichhagen J, Frank JA, Trauner D (2015). A roadmap to success in photopharmacology. Acc. Chem. Res..

[22] Erbas-Cakmak S, Leigh DA, McTernan CT, Nussbaumer AL (2015). Artificial molecular machines. Chem. Rev..

[23] Kay ER, Leigh DA, Zerbetto F (2007). Synthetic molecular motors and mechanical machines. Angew. Chem., Int. Ed..

[24] Hayasaka H, Miyashita T, Tamura K, Akagi K (2010). Helically π-stacked conjugated polymers bearing photoresponsive and chiral moieties in side chains: reversible photoisomerization-enforced switching between emission and quenching of circularly polarized fluorescence. Adv. Funct. Mater..

[25] Zhao D, van Leeuwen T, Cheng J, Feringa BL (2016). Dynamic control of chirality and self-assembly of double-stranded helicates with light. Nat. Chem..

[26] Qin L, Gu W, Wei J, Yu Y (2018). Piecewise phototuning of self-organized helical superstructures. Adv. Mater..

[27] Zheng Z-g (2016). Three-dimensional control of the helical axis of a chiral nematic liquid crystal by light. Nature.

[28] Pijper D, Jongejan MGM, Meetsma A, Feringa BL (2008). Light-controlled supramolecular helicity of a liquid crystalline phase using a helical polymer functionalized with a single chiroptical molecular switch. J. Am. Chem. Soc..

[29] Li, Q. *Photoactive Functional Soft Materials: Preparation, Properties, and Applications* (Wiley & Sons, 2018).

[30] Bisoyi HK, Li Q (2016). Light-driven liquid crystalline materials: from photo-induced phase transitions and property modulations to applications. Chem. Rev..

[31] Sud D, Norsten Tyler B, Branda Neil R (2005). Photoswitching of stereoselectivity in catalysis using a copper dithienylethene complex. Angew. Chem. Int. Ed..

[32] Yokoyama Y (2009). Chiral photochromism based on 6π-electrocyclization. New J. Chem..

[33] Nakagawa T, Ubukata T, Yokoyama Y (2018). Chirality and stereoselectivity in photochromic reactions. J. Photoch. Photobio. C: Photoch. Rev..

[34] Feringa BL, van Delden RA, Koumura N, Geertsema EM (2000). Chiroptical molecular switches. Chem. Rev..

[35] Petermayer C, Dube H (2018). Circular dichroism photoswitching with a twist: axially chiral hemiindigo. J. Am. Chem. Soc..

[36] Jurissek C, Berger F, Eisenreich F, Kathan M, Hecht S (2019). External reversal of chirality transfer in photoswitches. Angew. Chem., Int. Ed..

[37] Butterfield AM, Gilomen B, Siegel JS (2012). Kilogram-scale production of corannulene. Org. Process Res. Dev..

[38] Borchard, A., Hardcastle, K., Gantzel, P. & Siegel, J. S. 1,6,7,10-Tetramethylfluoranthene: synthesis and structure of a twisted polynuclear aromatic hydrocarbon. *Tetrahedron Lett.* **34**, 273–276 (1993).

[39] Borchardt, A., Fuchicello, A., Kilway, K. V., Baldridge, K. K. & Siegel, J. S. Synthesis and dynamics of the corannulene nucleus.* J. Am. Chem. Soc.* **114**, 1921–1923 (1992).

[40] Duda B, Lentz D (2015). Simultaneous introduction of trifluoromethyl and λ^6^-pentafluorosulfanyl substituents using F_5_S-C≡C-CF_3_ as a dienophile. Org. Biomol. Chem..

[41] Jones, D. W. & McDonald, W. S. Exceptional dimerisation of 7,9-dimethylcyclopent[*a*]acenaphthylen-8-one; X-ray crystal structure of the diol-diacetate of one of the dimeric products. *J. Chem. Soc. Chem. Commun*. **10**, 417–418 (1980).

[42] Allen FH (1987). Tables of bond lengths determined by X-ray and neutron diffraction. Part 1. Bond lengths in organic compounds. J. Chem. Soc. Perkin Trans..

[43] Johnson ER (2010). Revealing noncovalent interactions. J. Am. Chem. Soc..

[44] Houk KN, Northington DJ (1972). Photochemistry of cyclopentadienone dimers. Tetrahedron Lett..

[45] Fuchs B (1980). Thermal and photochemical transformation modes of cyclopentadienone-dimers: structural and stereochemical aspects. Isr. J. Chem..

[46] B. WR, Roald H (1969). The conservation of orbital symmetry. Angew. Chem., Int. Ed..

[47] Yamaguchi T, Kobayashi Y, Abe J (2016). Fast negative photochromism of 1,1’-binaphthyl-bridged phenoxyl-imidazolyl radical complex. J. Am. Chem. Soc..

[48] Hatano S, Horino T, Tokita A, Oshima T, Abe J (2013). Unusual negative photochromism via a short-lived imidazolyl radical of 1,1’-binaphthyl-bridged imidazole dimer. J. Am. Chem. Soc..

[49] Szymański W, Beierle JM, Kistemaker HAV, Velema WA, Feringa BL (2013). Reversible photocontrol of biological systems by the incorporation of molecular photoswitches. Chem. Rev..

[50] Iwaso K, Takashima Y, Harada A (2016). Fast response dry-type artificial molecular muscles with [c2]daisy chains. Nat. Chem..

[51] Huang S-L, Hor TSA, Jin G-X (2017). Photodriven single-crystal-to-single-crystal transformation. Coord. Chem. Rev..

[52] Moth-Poulsen K, Bjornholm T (2009). Molecular electronics with single molecules in solid-state devices. Nat. Nanotechnol..

[53] Frisenda R, Janssen VAEC, Grozema FC, van der Zant HSJ, Renaud N (2016). Mechanically controlled quantum interference in individual π-stacked dimers. Nat. Chem..

[54] Stern HL (2017). Vibronically coherent ultrafast triplet-pair formation and subsequent thermally activated dissociation control efficient endothermic singlet fission. Nat. Chem..

[55] Smith MB, Michl J (2010). Singlet fission. Chem. Rev..

[56] Zhang J (2017). Remote light-controlled intracellular target recognition by photochromic fluorescent glycoprobes. Nat. Commun..

[57] Samanta D (2018). Reversible chromism of spiropyran in the cavity of a flexible coordination cage. Nat. Commun..

[58] Li R-J, Holstein JJ, Hiller WG, Andréasson J, Clever GH (2019). Mechanistic interplay between light switching and guest binding in photochromic [Pd_2_dithienylethene_4_] coordination cages. J. Am. Chem. Soc..

