# Author Correction: Unveiling how intramolecular stacking modes of covalently linked dimers dictate photoswitching properties

**DOI:** 10.1038/s41467-020-15256-2

**Published:** 2020-03-31

**Authors:** Ru-Qiang Lu, Xiao-Yun Yan, Lei Zhu, Lin-Lin Yang, Hang Qu, Xin-Chang Wang, Ming Luo, Yu Wang, Rui Chen, Xiao-Ye Wang, Yu Lan, Jian Pei, Wengui Weng, Haiping Xia, Xiao-Yu Cao

**Affiliations:** 1 State Key Laboratory of Physical Chemistry of Solid Surfaces, Collaborative Innovation Center of Chemistry for Energy Materials (iChEM), Key Laboratory for Chemical Biology of Fujian Province, Department of Chemistry, College of Chemistry and Chemical Engineering, Xiamen University, 361005 Xiamen, China; 2 Department of Polymer Science, College of Polymer Science and Polymer Engineering, The University of Akron, Akron, OH 44325-3909 USA; 3 School of Chemistry and Chemical Engineering, Chongqing University, 400030 Chongqing, China; 4 State Key Laboratory of Elemento-Organic Chemistry, College of Chemistry, Nankai University, 300071 Tianjin, China; 5 Beijing National Laboratory for Molecular Sciences (BNLMS), the Key Laboratory of Bioorganic Chemistry and Molecular Engineering of Ministry of Education, College of Chemistry and Molecular Engineering, Peking University, 100871 Beijing, China

**Keywords:** Optical materials, Organic chemistry, Photochemistry

Correction to: *Nature Communications* 10.1038/s41467-019-13428-3, published online 02 December 2019.

The original version of this Article contained an error in Fig. 5, in which the incorrect structure was shown for compound **4c**. The correct version of Fig. 5 is:



which replaces the previous incorrect version:



This has been corrected in both the PDF and HTML versions of the Article.

