# Supplementary material for: Unveiling how intramolecular stacking modes of covalently linked dimers dictate photoswitching properties
Source: Nat Commun. 2019 Dec 2;10:5480. doi: 10.1038/s41467-019-13428-3 (PMC6889182; doi:10.1038/s41467-019-13428-3)
Supplement: Supplementary file 1 — Supplementary Information [file 41467_2019_13428_MOESM1_ESM.pdf]

Supplementary Information

Unveiling How Intramolecular Stacking Modes of Covalently Linked Dimers Dictate Photoswitching Properties

by Lu and Yan et al.

Supplementary Figures

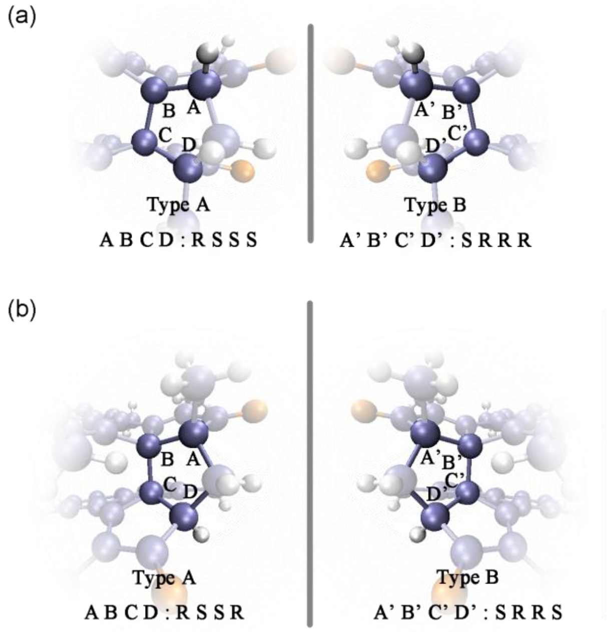

Supplementary Figure 1. X-ray single-crystal structures showing the enantiomers of (a) **3a** and (b) **3b**.

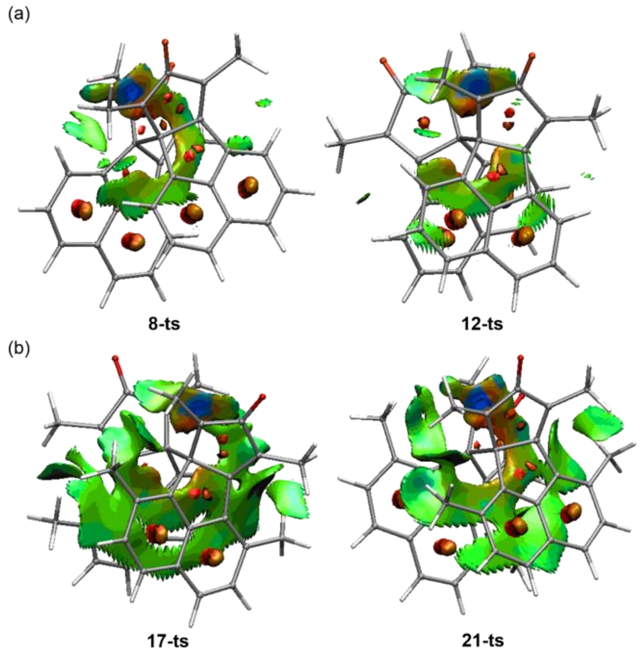

Supplementary Figure 2. Non-covalent interaction (NCI) analysis for (a) **8-ts** and **12-ts** and (b) **17-ts** and **21-ts** (blue, strong attraction; green, weak attractive interaction; red, steric effect).

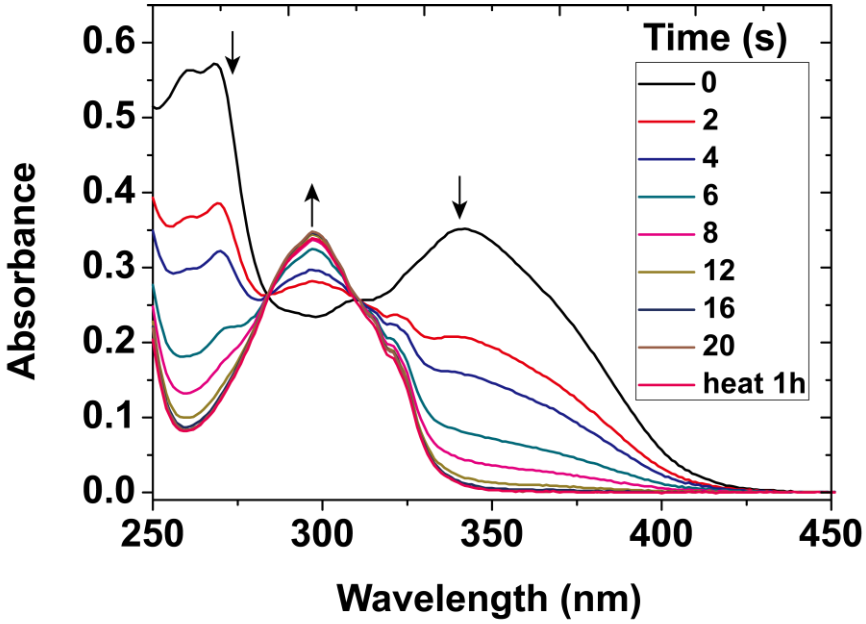

Supplementary Figure 3. Absorption spectra of **3a** upon UV irradiation at 365 nm in $CHCl_3$ (2.0 × 10^{-5} M). The photochemical [2+2] cycloaddition was finished completely in less than 16 s. The cycloaddition product **4a** was stable upon heating for 1 h.

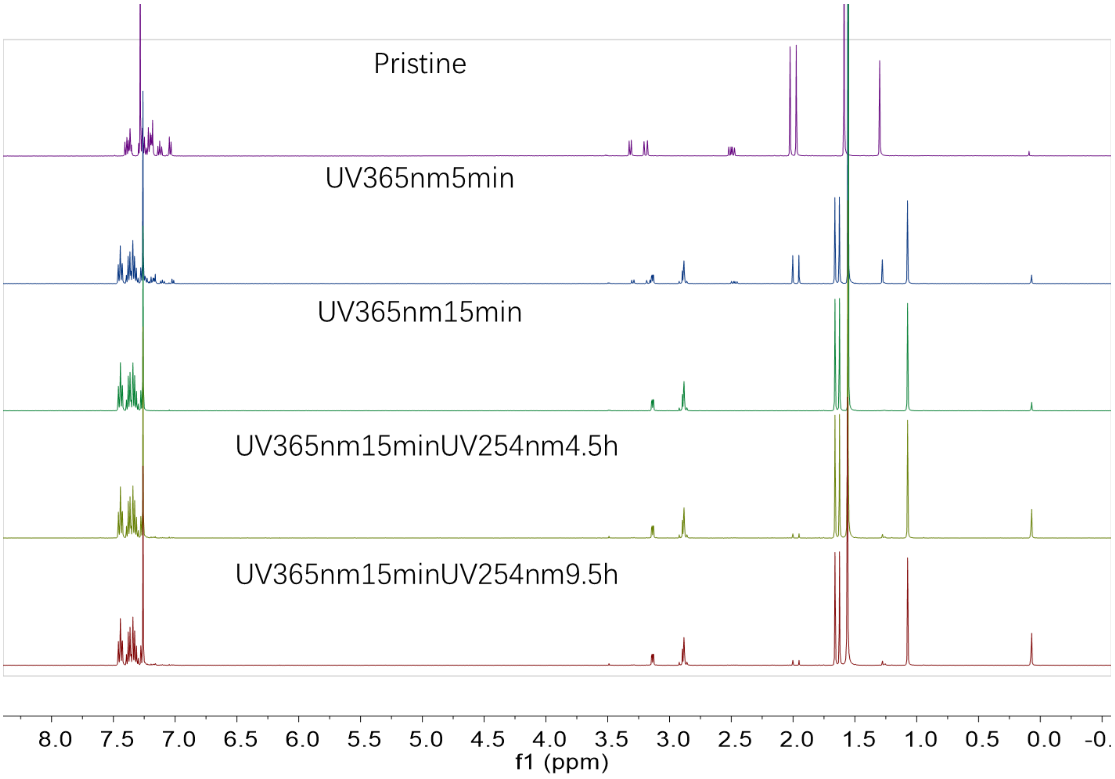

Supplementary Figure 4. ^{1}H NMR spectra of **3a** (5.85 mM in $CDCl_3$, 500 MHz) in the pristine state, and after UV irradiation at 365 nm for 5 min and 15 min, and then after UV irradiation at 254 nm for 4.5 h and 9.5 h.

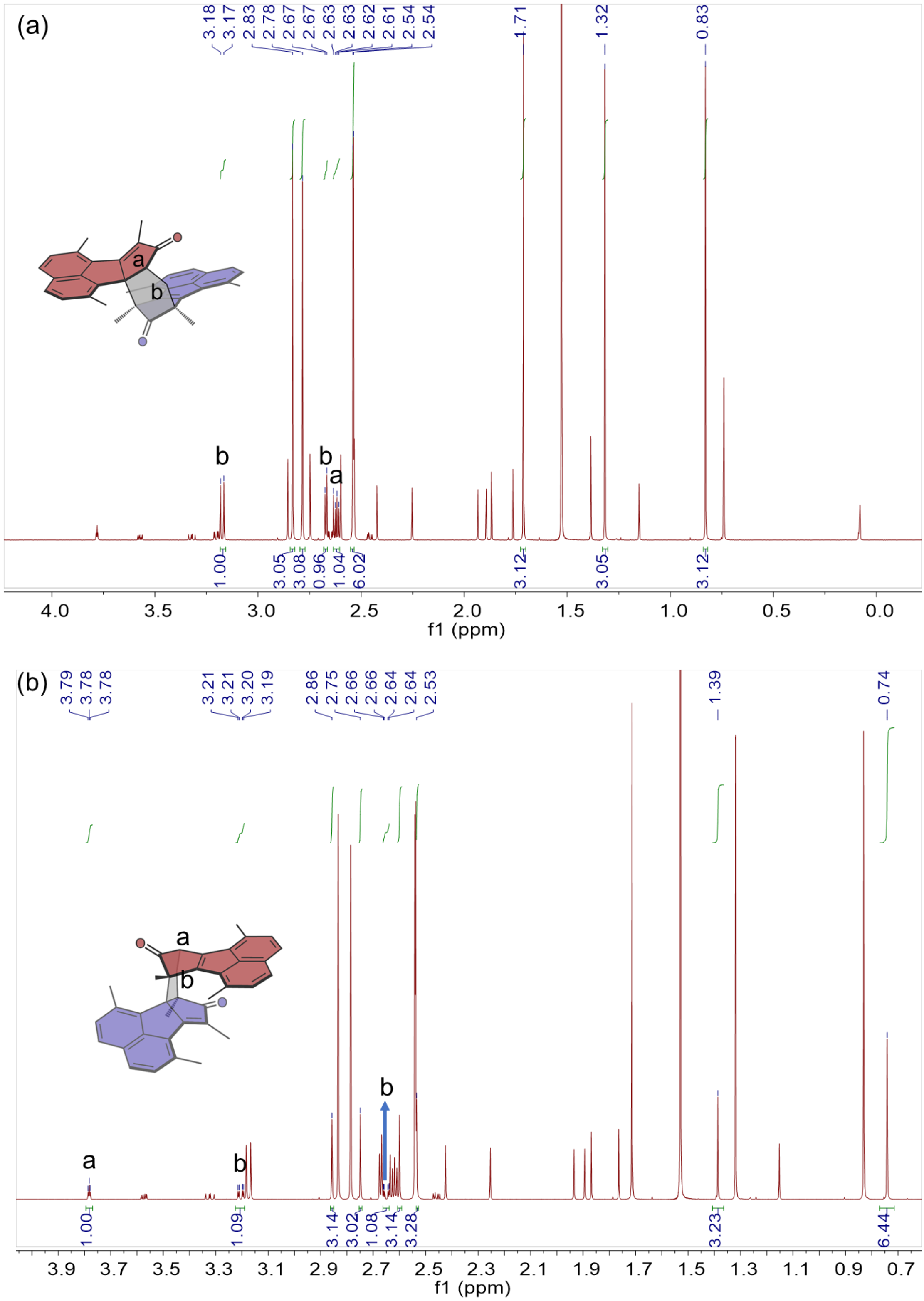

Supplementary Figure 5. ^{1}H NMR spectra of the mixture of **3b, 4b** and **4c** in the upfield region ($CDCl_3$, 850 MHz). (a) The chemical shifts and integrals of the protons of **4b**. (b) The chemical shifts and integrals of the protons of **4c**. The number and chemical shifts of protons were in accordance with the structures of **4b** and **4c**.

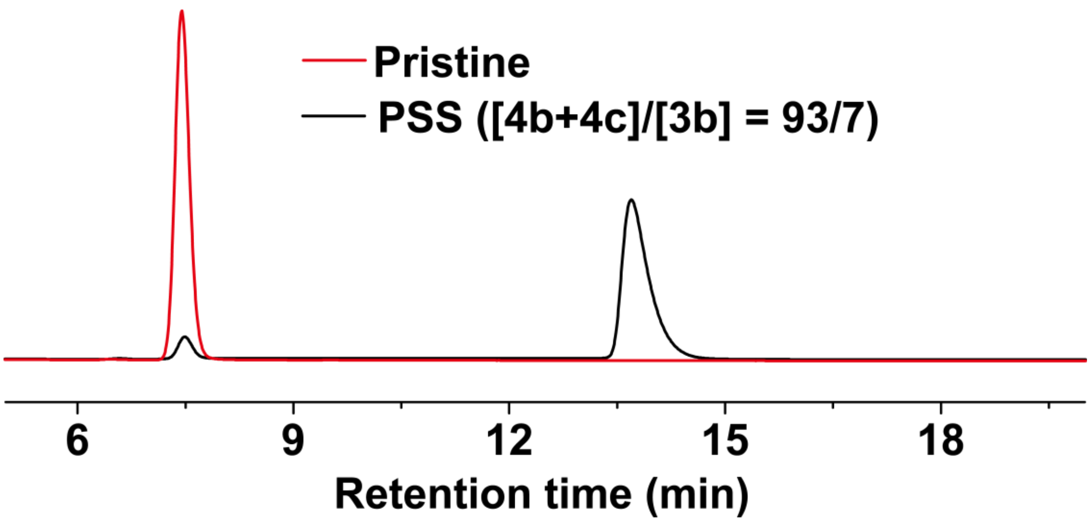

Supplementary Figure 6. Determination of the photostationary state (PSS) of **3b** (0.961 mM in CH_2Cl_2) by HPLC spectra (column: ODS column, eluent: MeOH, temperature: 301 K, flow rate: 0.7 mL·min^{-1}; the monitor wavelength is at the isobestic point: 300 nm).

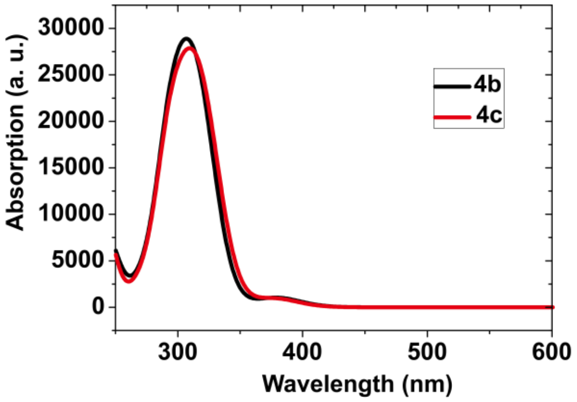

Supplementary Figure 7. Calculated UV-vis spectra of **4b** and **4c** (TD-DFT calculations at the ωB97X-D/6-31G(d,p) level of theory in the gas phase).

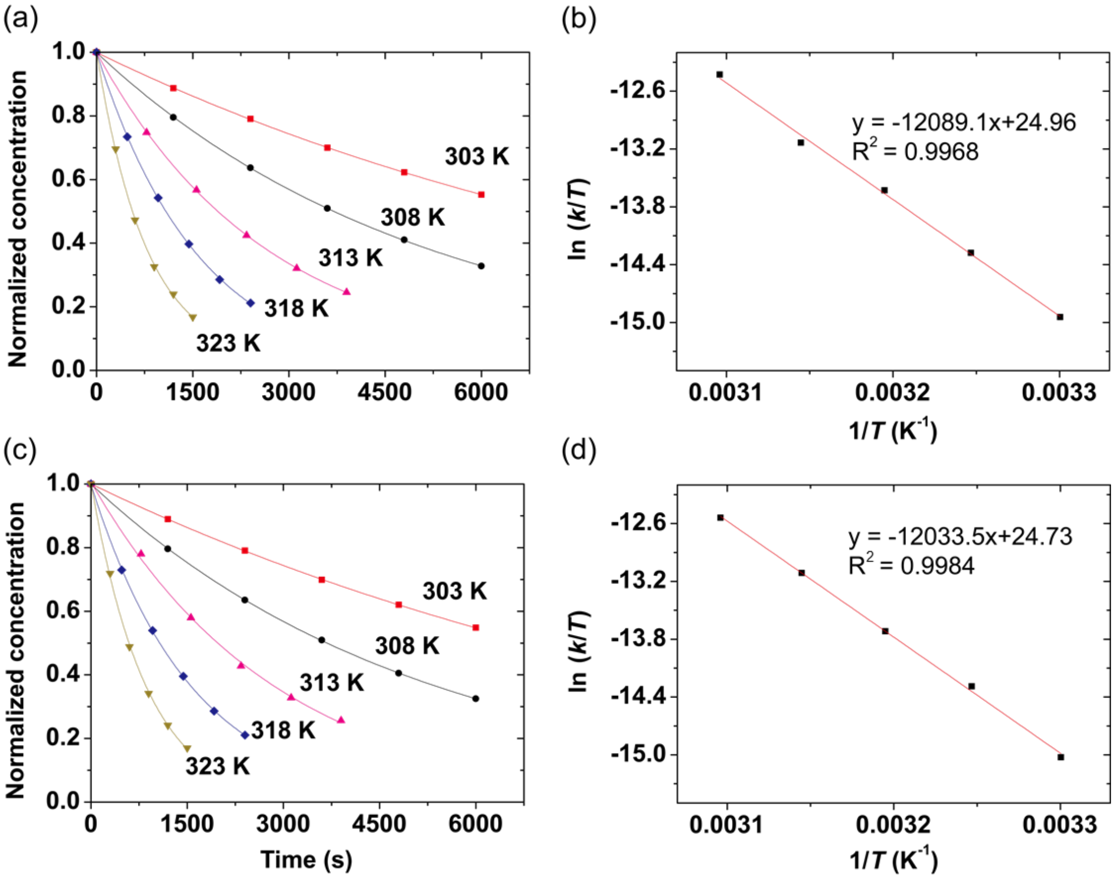

Supplementary Figure 8. (a) and (c) time-dependent concentration of **4b** and **4c** at various temperatures (solvent: $CDCl_3$). (b) and (d) rate constants at different temperatures fitted by the Eyring equation ($\ln(k/T) = -\Delta H^{\ddagger}/(RT) + \ln(k_B/h) + \Delta S^{\ddagger}/R$, where $\Delta H^{\ddagger}$ is the activation enthalpy, R is the gas constant, T is the temperature, k_B is the Boltzmann constant, h is the Planck constant, and $\Delta S^{\ddagger}$ is the activation entropy). The activation enthalpy and activation entropy were determined as 100.5 kJ·mol^{-1} and –9.98 J·(mol·K)$^{-1}$ for the thermal isomerization from **4b** to **3b**, and 100.0 kJ·mol^{-1} and –8.06 J·(mol·K)$^{-1}$ for the thermal isomerization from **4c** to **3b**.

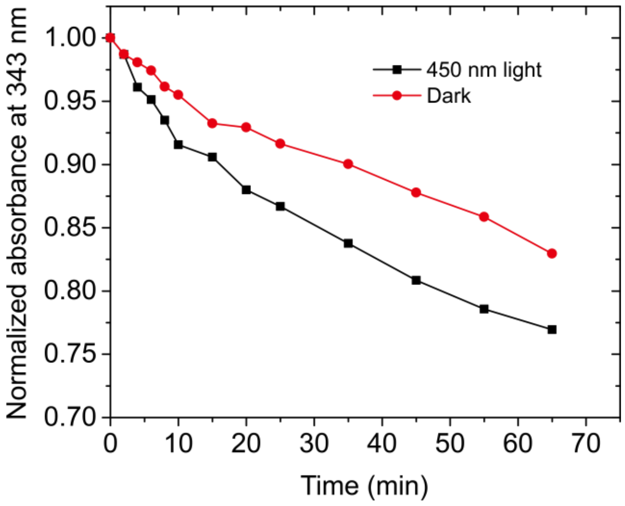

Supplementary Figure 9. Time-dependent absorbance of photostationary state (PSS) solution of **3b** at 343 nm in dark and upon visible light irradiation at λ = 450 nm. The solution of **3b** in $CHCl_3$ (2.0 mM) was irradiated with UV light (λ = 365 nm) to the PSS and used immediately. An acceleration of the back-switching is observed, revealing the back-switching can be triggered by both heat and visible light.

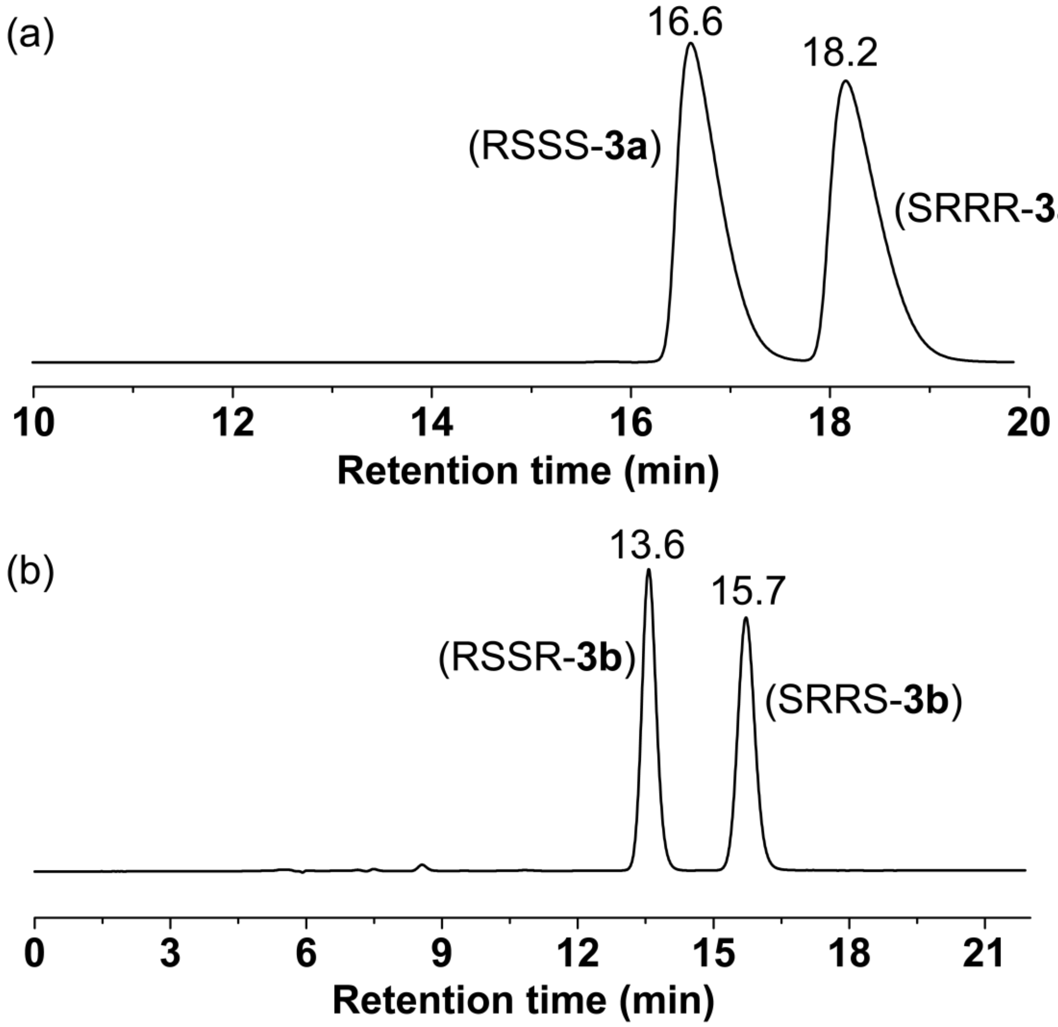

Supplementary Figure 10. (a) The chiral HPLC (column: Daicel Chiralcel IE, flow rate: 0.8 mL·min^{-1}) spectrum of **3a** eluting with $CHCl_3$. The portion with the retention time at 16.6 min corresponds to RSSS-**3a**. The portion with the retention time at 18.2 min corresponds to SRRR-**3a**. (b) The chiral HPLC (column: Daicel Chiralcel IC, flow rate: 0.6 mL·min^{-1}) spectrum of **3b** eluting with $CHCl_3$/ethyl acetate (*v*/*v*: 70/30). The portion with the retention time at 13.6 min corresponds to RSSR-**3b**. The portion with the retention time at 15.7 min corresponds to SRRS-**3b**.

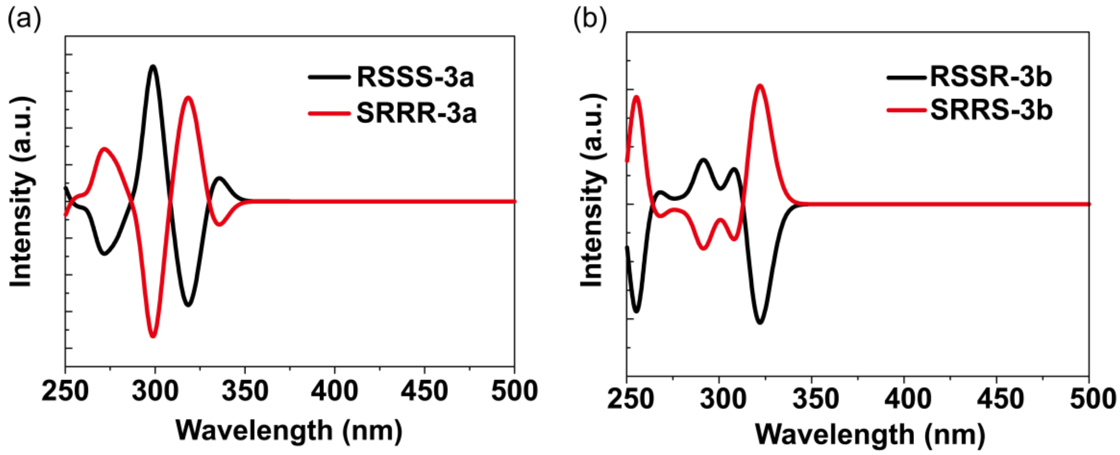

Supplementary Figure 11. Calculated CD spectra of (a) **3a** and (b) **3b** (TD-DFT calculations at the ωB97X-D/6-31G(d,p) level of theory in the gas phase).

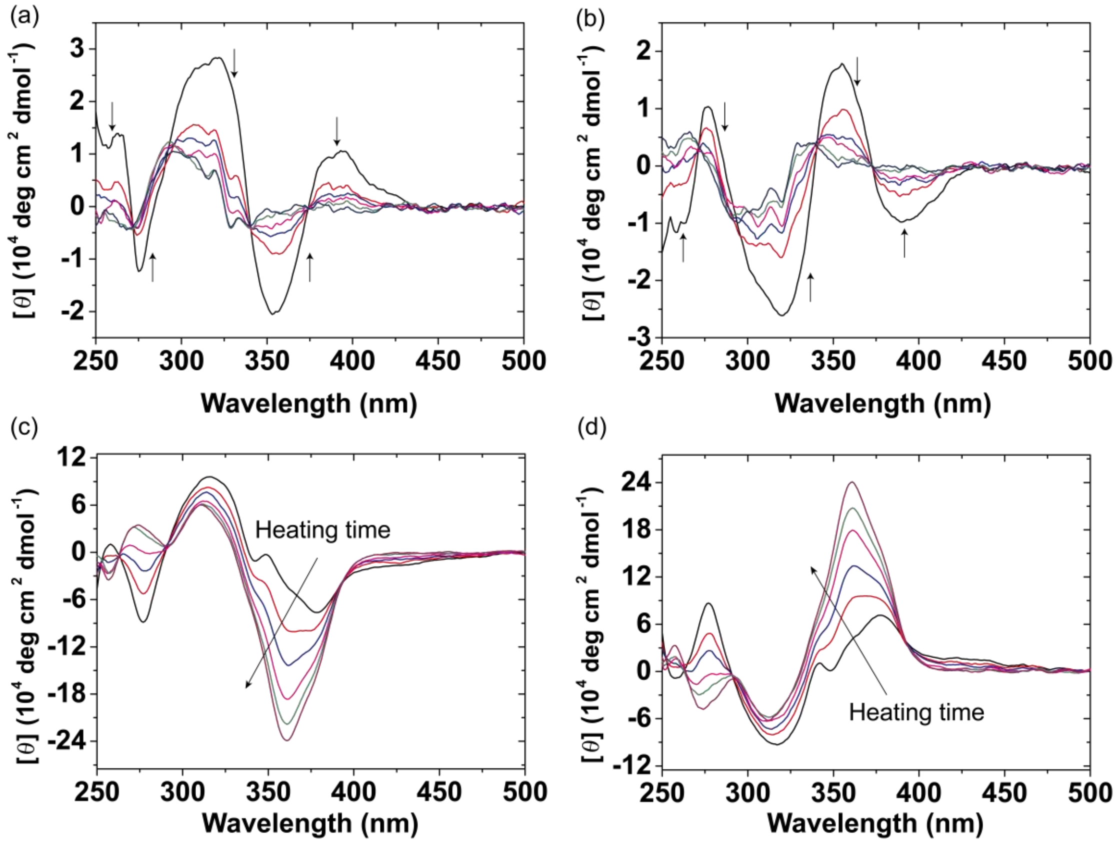

Supplementary Figure 12. CD spectra of (a) RSSS-**3a** and (b) SRRR-**3a** upon UV irradiation (λ = 365 nm). CD spectra of (c) RSSR-**3b** and (d) SRRS-**3b** in the photostationary states upon heating at 55 °C.

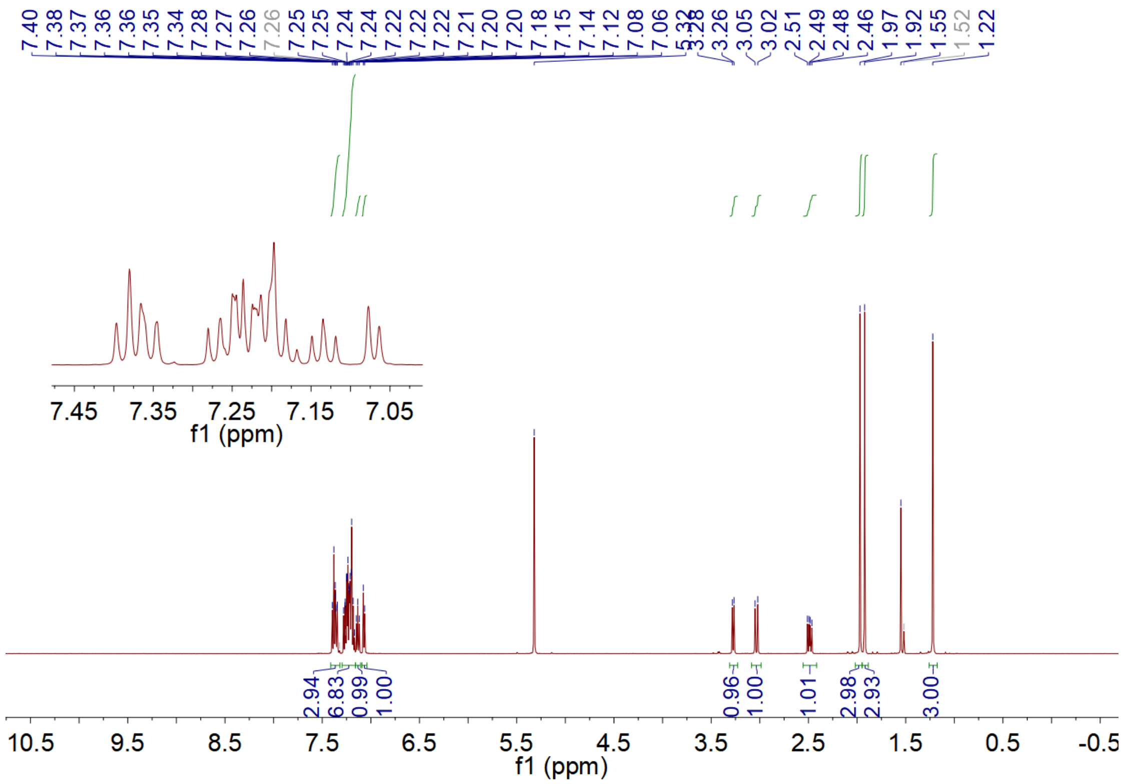

Supplementary Figure 13. ^{1}H NMR spectrum of **3a** (500 M, CD_2Cl_2)

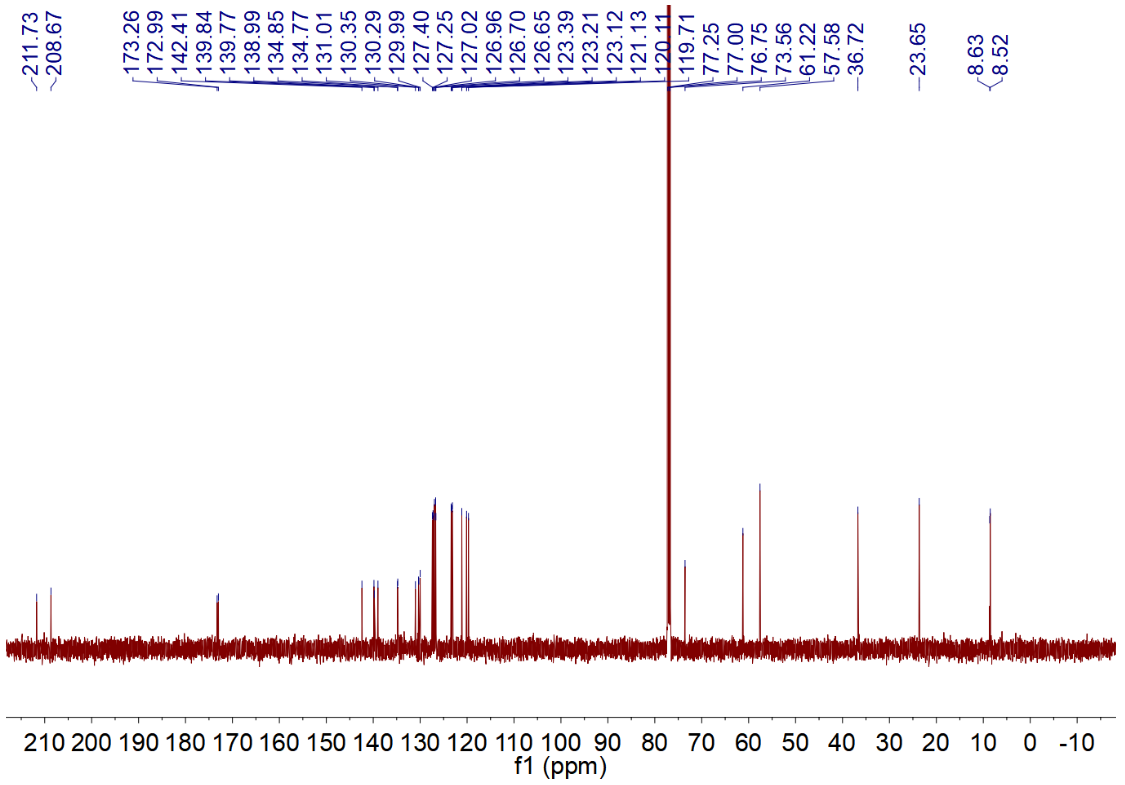

Supplementary Figure 14. ^{13}C NMR spectrum of **3a** (126 M, $CDCl_3$)

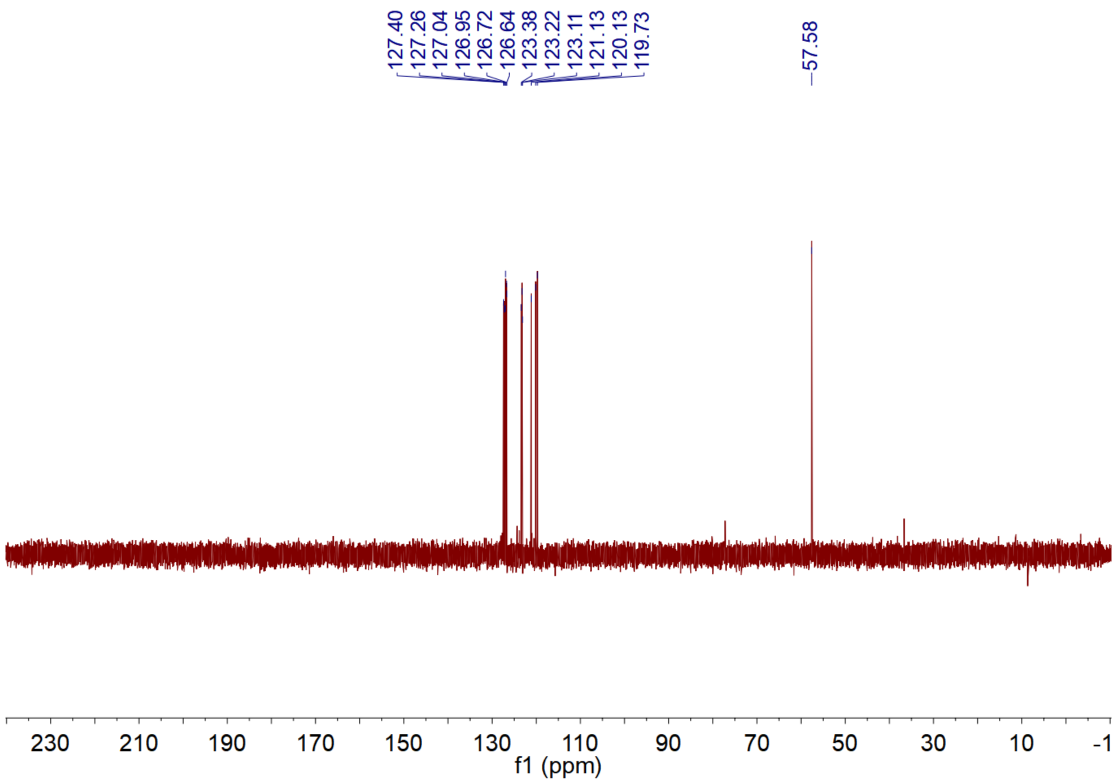

Supplementary Figure 15. Distortionless enhancement by polarization transfer 90 (DEPT90) spectrum of **3a** (214 M, $CDCl_3$)

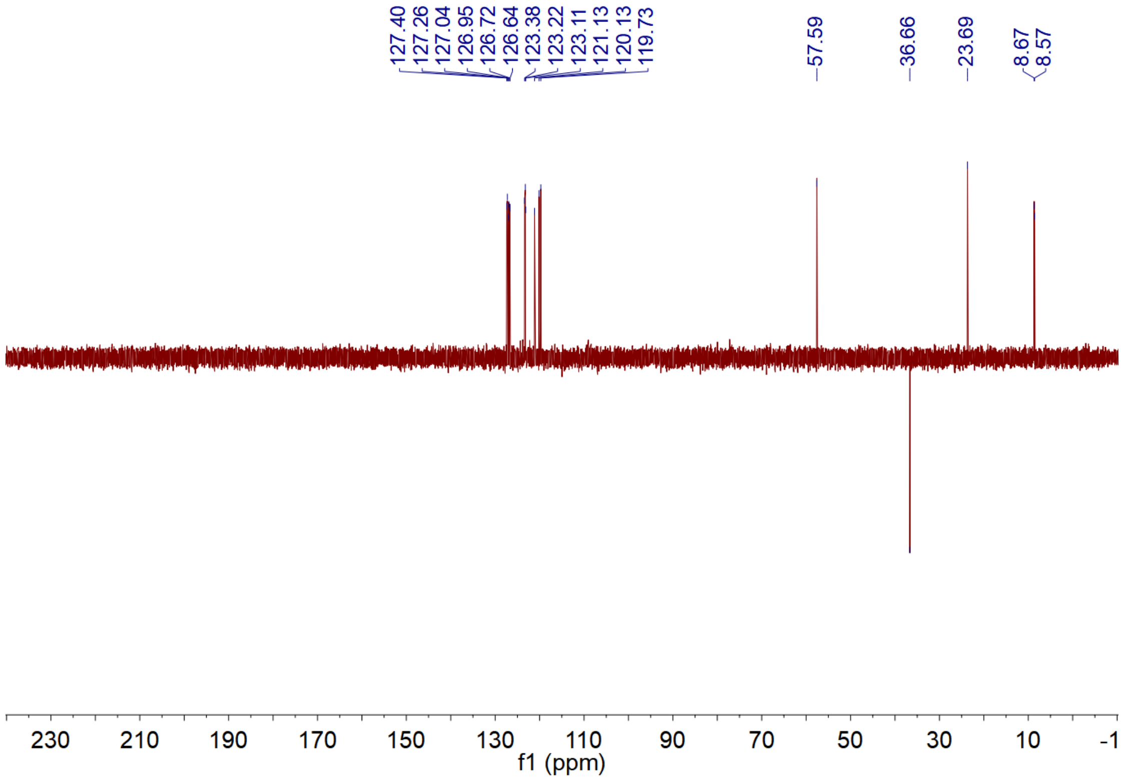

Supplementary Figure 16. DEPT135 spectrum of **3a** (214 M, $CDCl_3$)

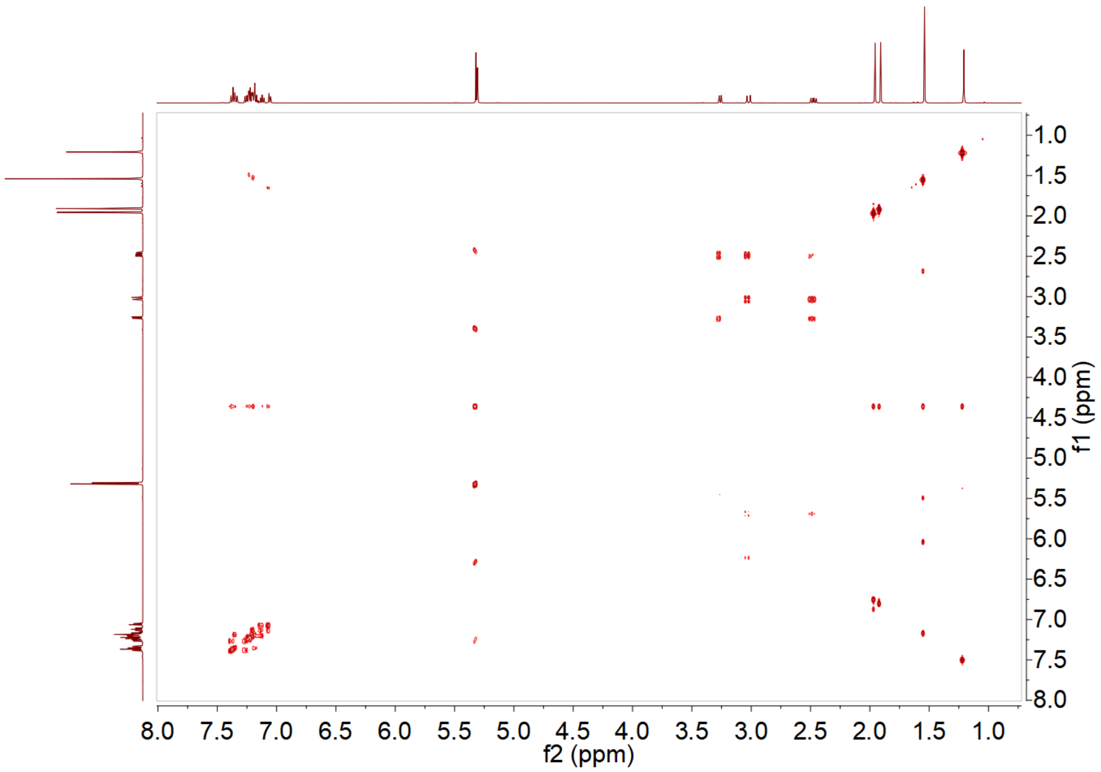

Supplementary Figure 17. Correlation spectroscopy (COSY) spectrum of **3a** (500 M, CD_2Cl_2)

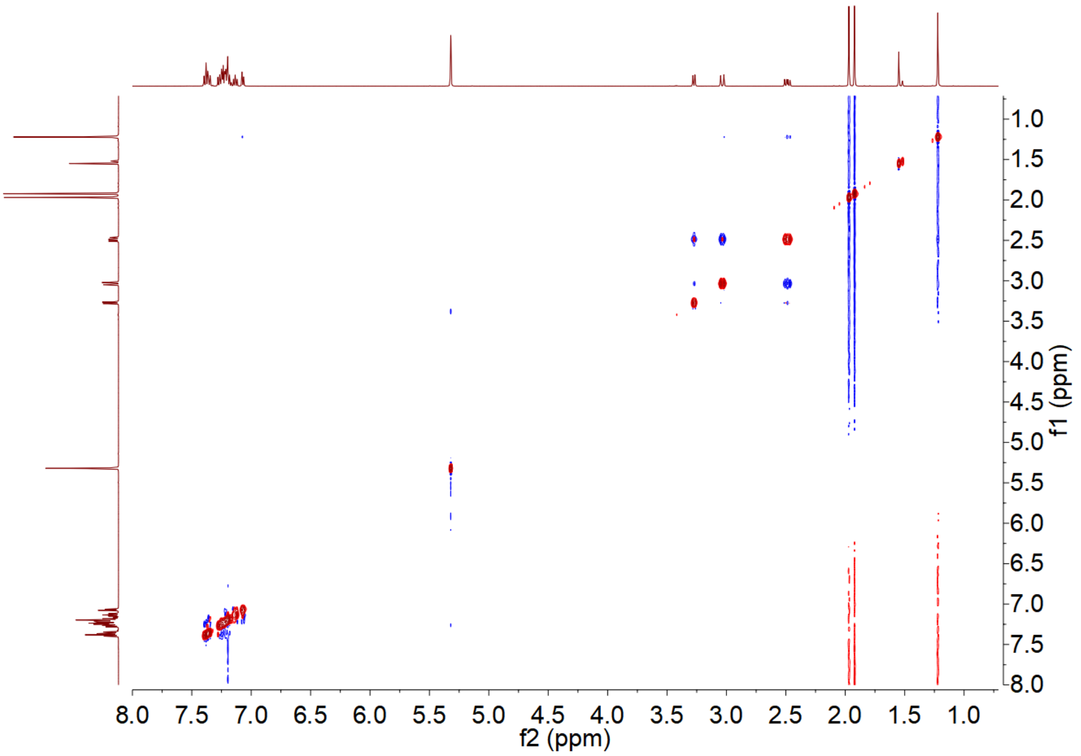

Supplementary Figure 18. Nuclear Overhauser enhancement spectroscopy (NOESY) spectrum of **3a** (500 M, CD_2Cl_2)

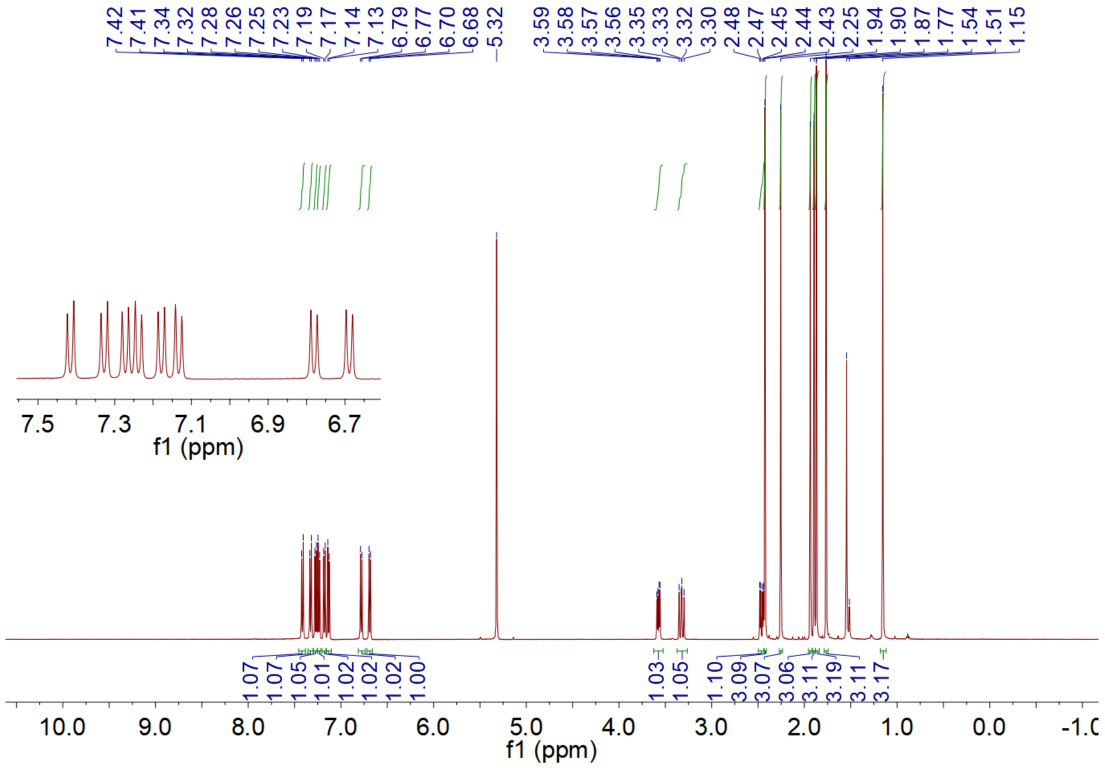

Supplementary Figure 19. ^{1}H NMR spectrum of **3b** (500M, CD_2Cl_2)

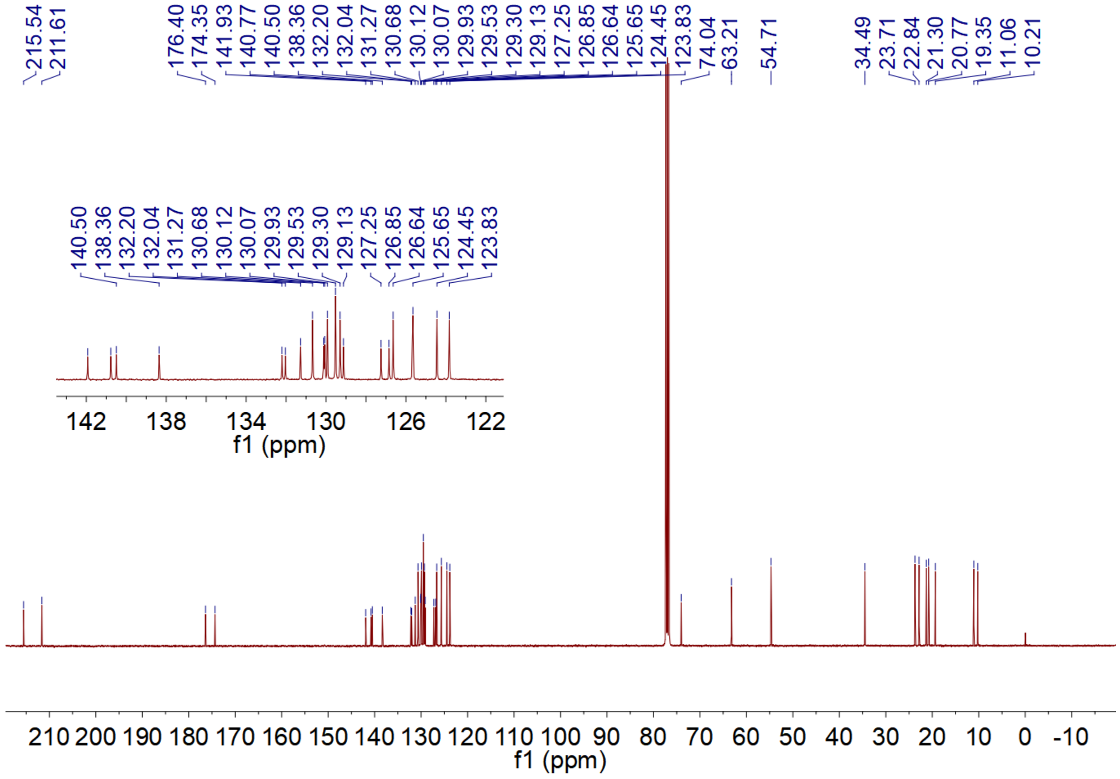

Supplementary Figure 20. ^{13}C NMR spectrum of **3b** (101 M, $CDCl_3$)

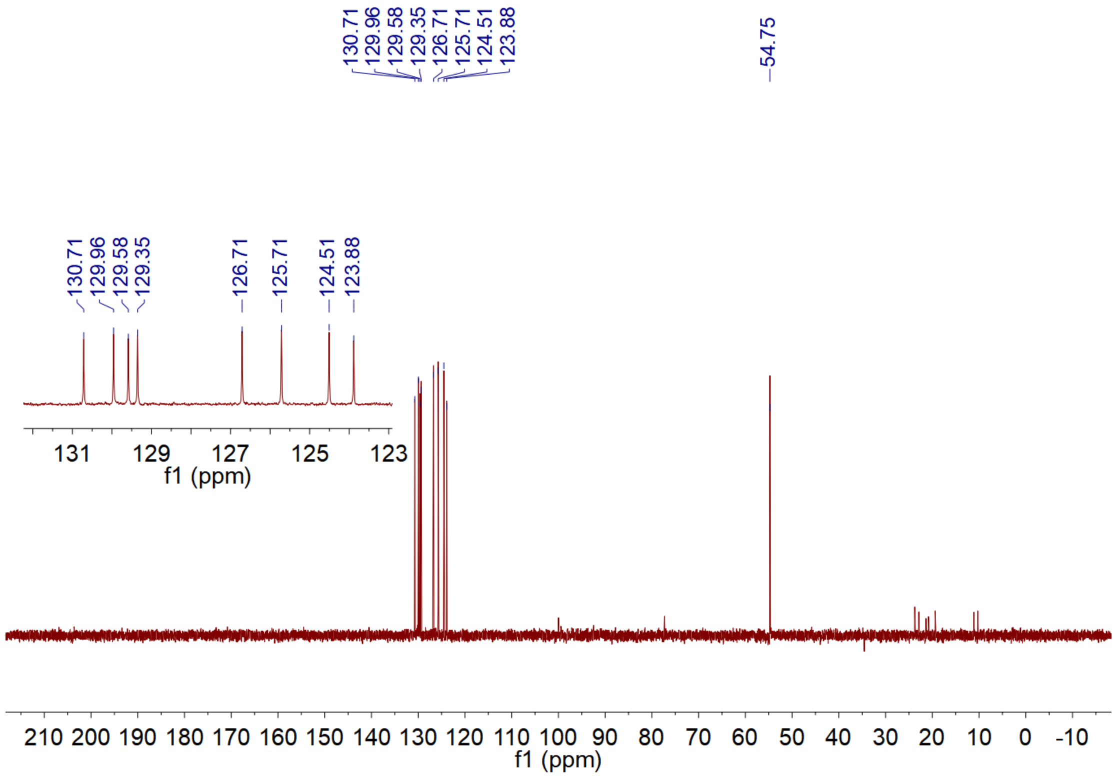

Supplementary Figure 21. DEPT90 spectrum of **3b** (126 M, $CDCl_3$)

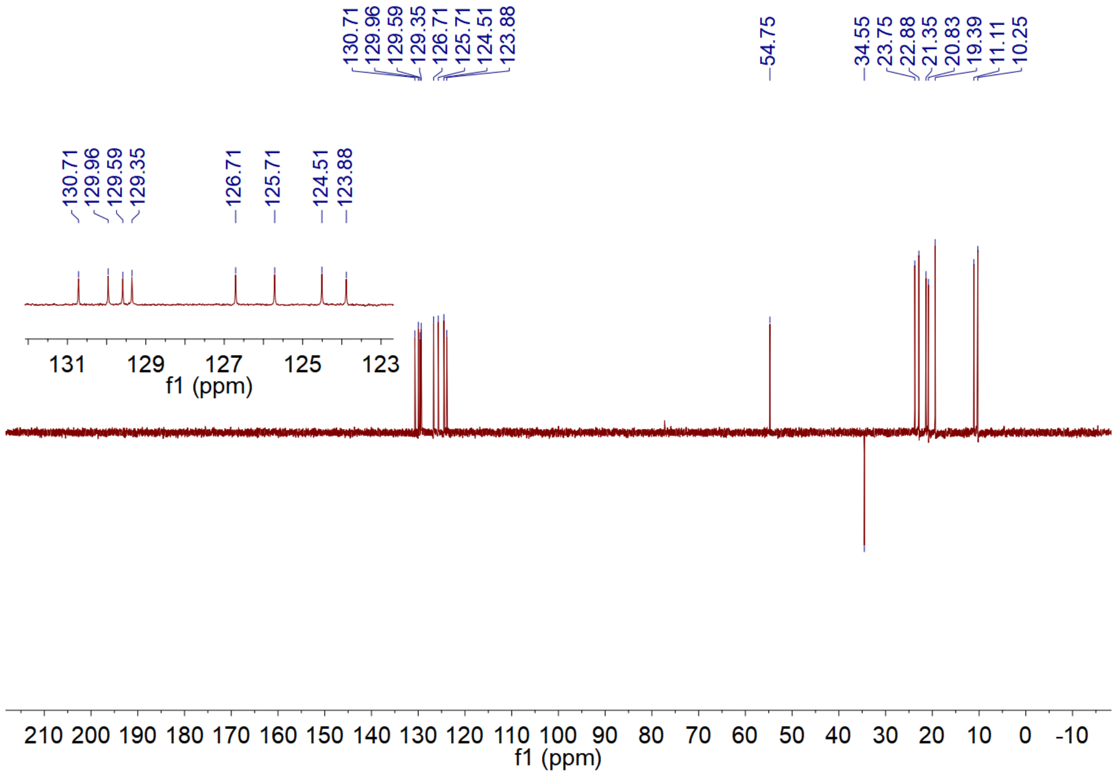

Supplementary Figure 22. DEPT135 spectrum of **3b** (126 M, $CDCl_3$)

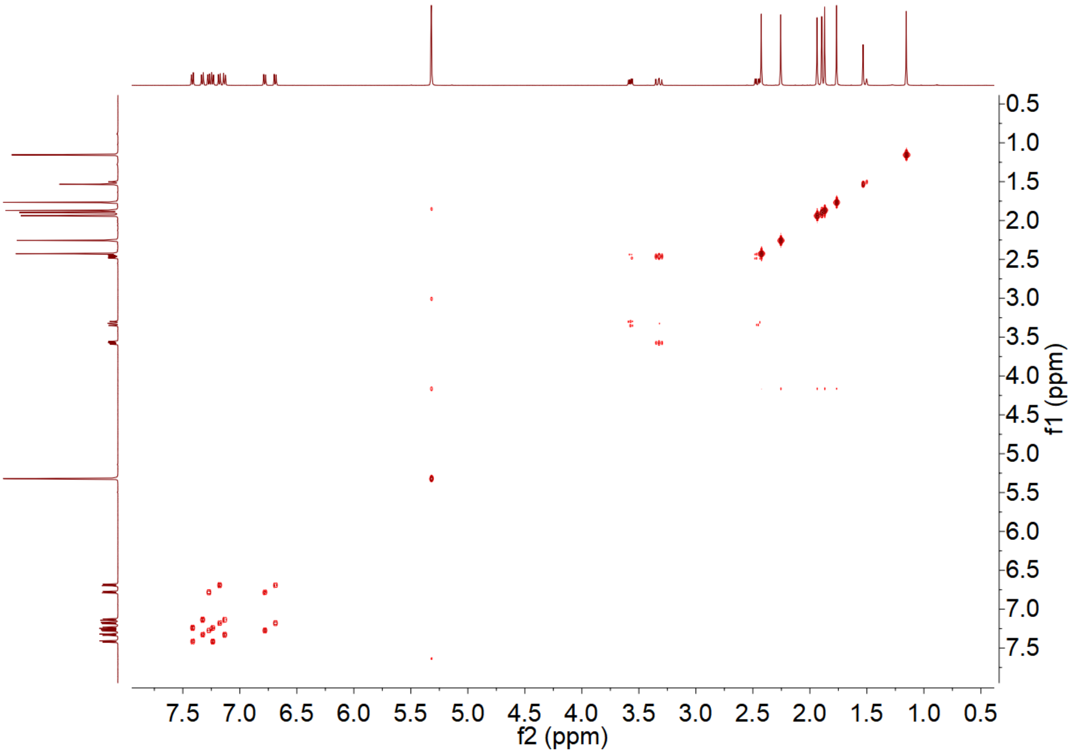

Supplementary Figure 23. COSY spectrum of **3b** (500 M, CD_2Cl_2)

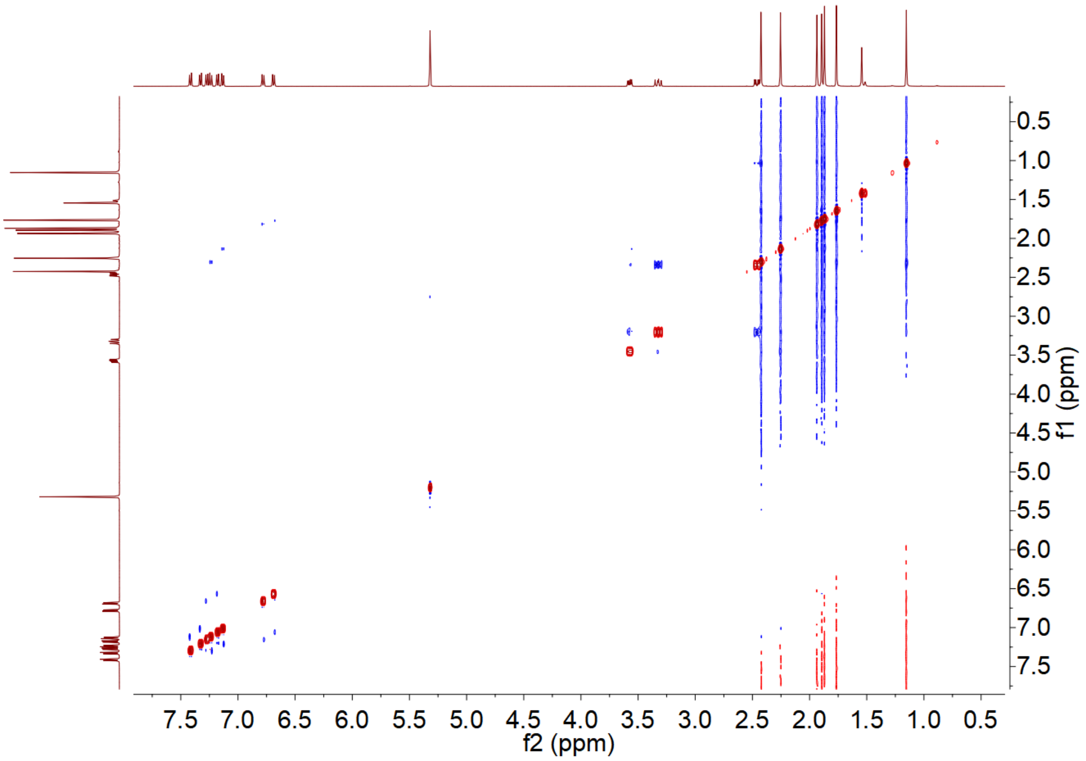

Supplementary Figure 24. NOESY spectrum of **3b** (500 M, CD_2Cl_2)

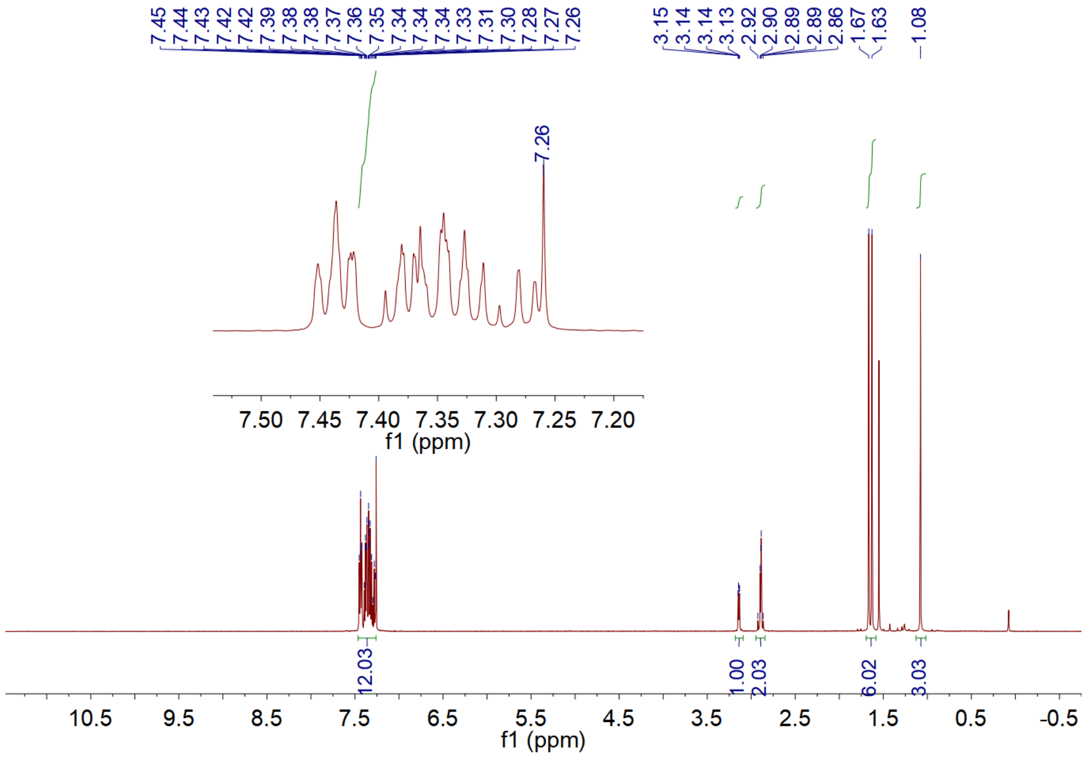

Supplementary Figure 25. ^{1}H NMR spectrum of **4a** (500 M, $CDCl_3$)

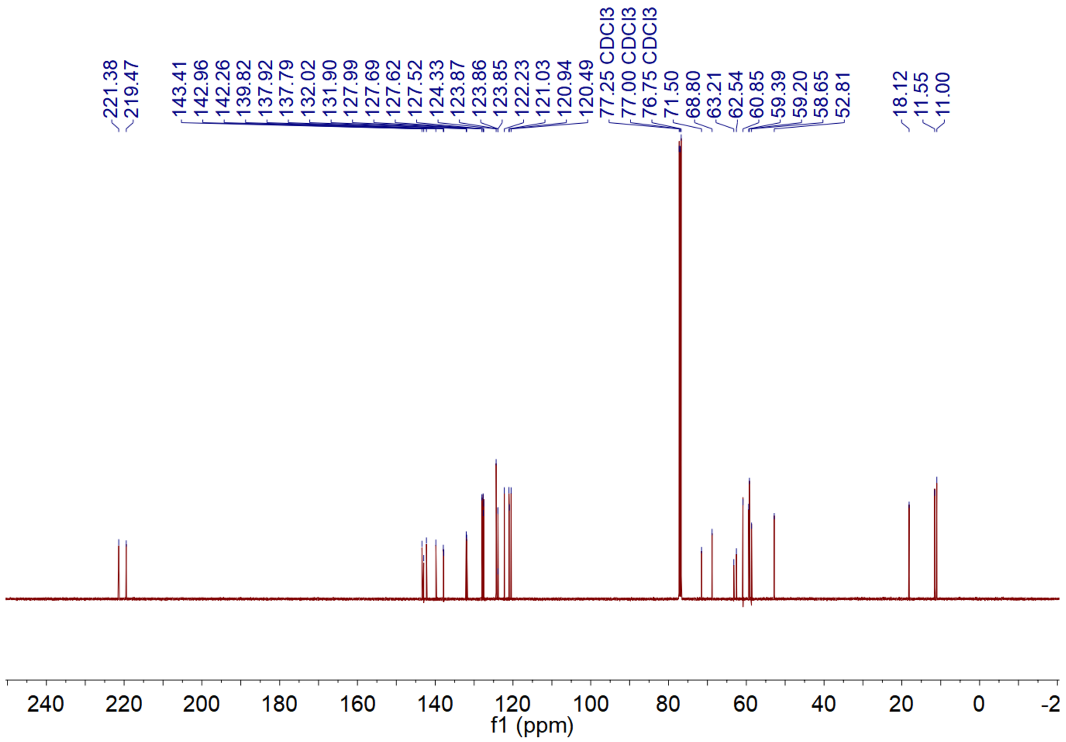

Supplementary Figure 26. ^{13}C NMR spectrum of **4a** (126 M, $CDCl_3$)

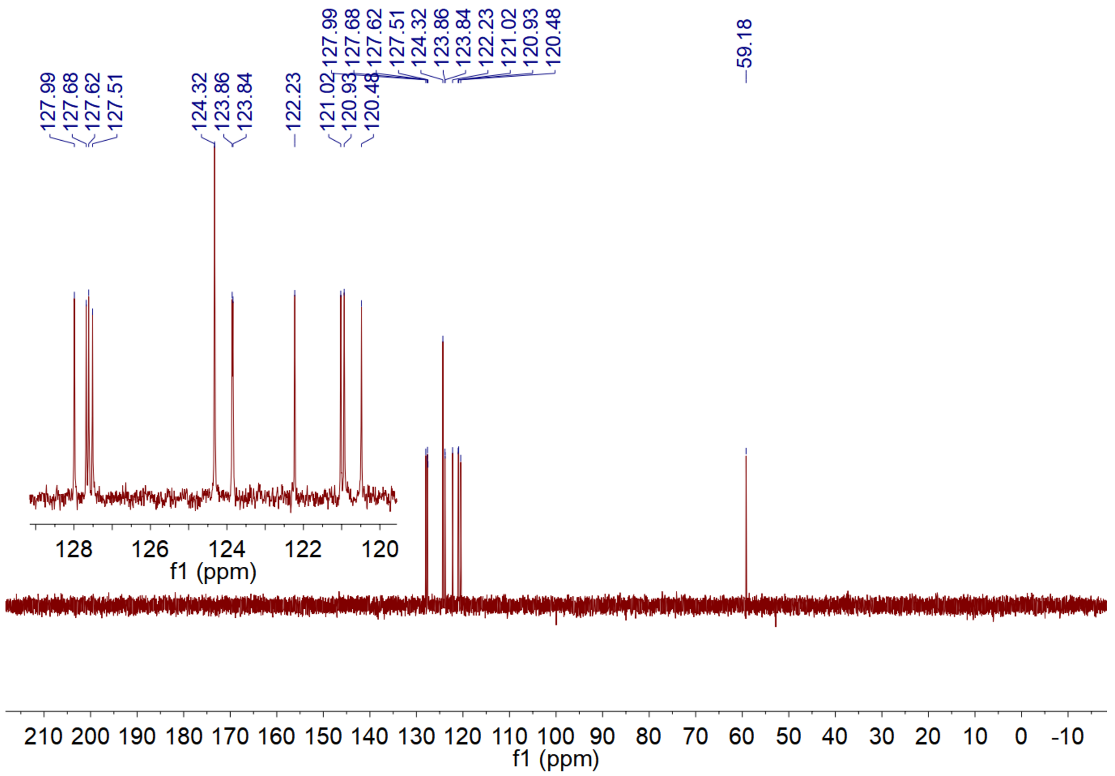

Supplementary Figure 27. DEPT90 spectrum of **4a** (126 M, $CDCl_3$)

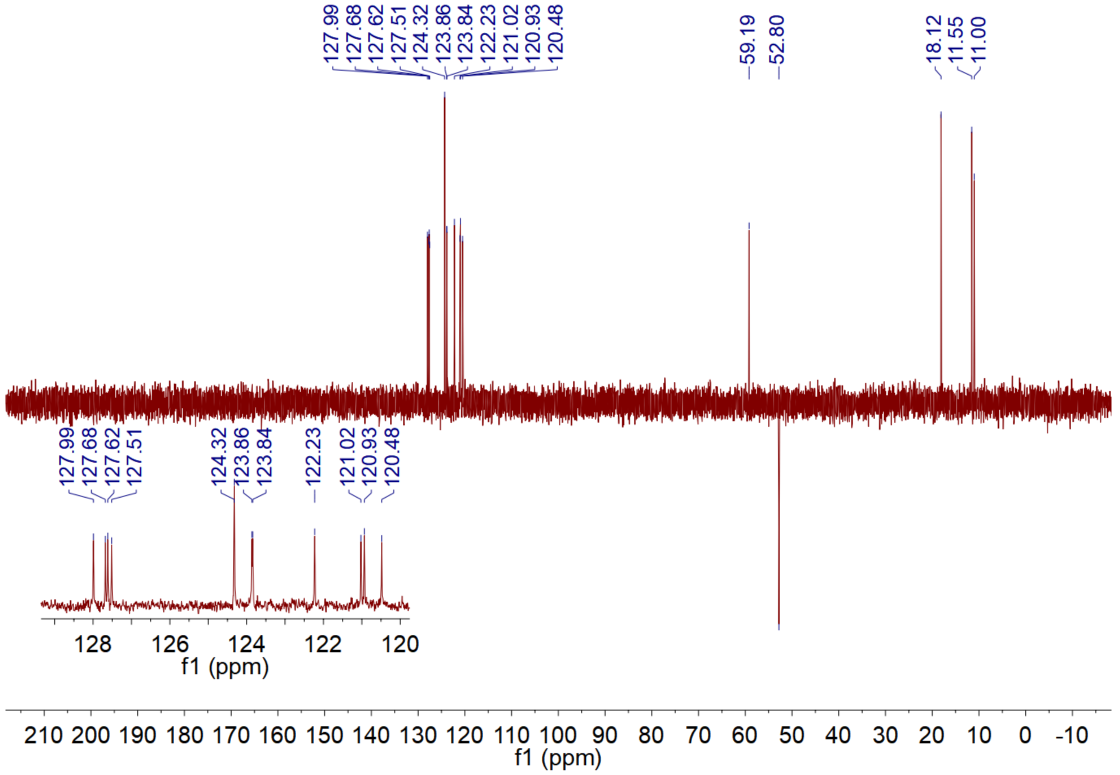

Supplementary Figure 28. DEPT135 spectrum of **4a** (126 M, $CDCl_3$)

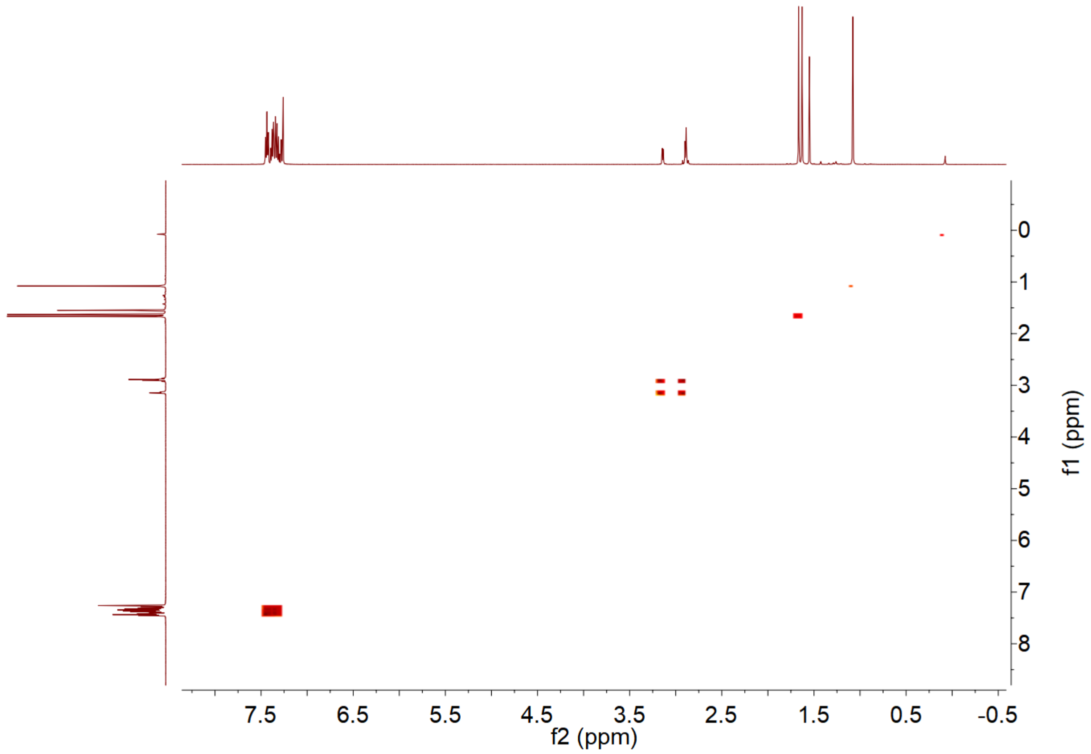

Supplementary Figure 29. COSY spectrum of **4a** (500 M, $CDCl_3$).

Supplementary Tables

Supplementary Table 1. Crystal data and structure refinements for **3a**, **3b**, **4a**, and **4b**.

Identification code	**3a**	**3b**	**4a**	**4b.2CH$_3$OH**
CCDC	1913518	1939142	1832043	1832044
Empirical formula	$C_{34}H_{24}O_2$	$C_{38}H_{32}O_2$	$C_{34}H_{24}O_2$	$C_{40}H_{40}O_4$
Formula weight	464.53	520.63	464.53	584.72
Temperature/K	100.00(10)	100.00(10)	100.00(10)	150.00(10)
Crystal system	monoclinic	orthorhombic	monoclinic	monoclinic
Space group	$P2_1/n$	$Pna2_1$	$P2_1/n$	$P2_1/c$
a/Å	9.70730(10)	20.4885(2)	10.0552(3)	10.6812(3)
b/Å	13.23450(10)	7.80430(10)	13.4897(3)	20.6208(7)
c/Å	18.2836(2)	16.39990(10)	17.4446(4)	13.9892(5)
α/°	90	90	90	90
β/°	97.6790(10)	90	96.321(2)	100.194(3)
γ/°	90	90	90	90
Volume/Å^3	2327.85(4)	2622.32(5)	2351.83(10)	3032.54(19)
Z	4	4	4	4
ρ_{calc}/g/cm^3	1.325	1.319	1.312	1.281
μ/mm^{-1}	0.632	0.617	0.626	0.638
F(000)	976.0	1104.0	976.0	1248.0
Crystal size/mm^3	0.2 × 0.2 × 0.15	0.2 × 0.15 × 0.12	0.2 × 0.2 × 0.15	0.2 × 0.1 × 0.1
Radiation	CuKα (λ = 1.54184)	CuKα (λ = 1.54184)	CuKα (λ = 1.54184)	CuKα (λ = 1.54184)
2Θ range for data collection/°	8.274 to 155.92	8.632 to 155.356	8.304 to 155.582	7.72 to 148.542
Index ranges	-12 ≤ h ≤ 12, -16 ≤ k ≤ 16, -23 ≤ l ≤ 16	-25 ≤ h ≤ 24, -9 ≤ k ≤ 9, -20 ≤ l ≤ 19	-12 ≤ h ≤ 12, -16 ≤ k ≤ 17, -21 ≤ l ≤ 12	-13 ≤ h ≤ 13, -25 ≤ k ≤ 25, -8 ≤ l ≤ 16
Reflections collected	32685	29513	28516	15282
Independent reflections	4924 [R_{int} = 0.0454, R_{sigma} = 0.0261]	5309 [R_{int} = 0.0516, R_{sigma} = 0.0344]	4931 [R_{int} = 0.0370, R_{sigma} = 0.0201]	5961 [R_{int} = 0.0521, R_{sigma} = 0.0553]
Data/restraints/parameters	4924/0/338	5309/1/368	4931/0/338	5961/0/409
Goodness-of-fit on F^2	1.064	1.071	1.113	1.106
Final R indexes [I>=2σ (I)]	R_1 = 0.0907, wR_2 = 0.2154	R_1 = 0.0341, wR_2 = 0.0855	R_1 = 0.0834, wR_2 = 0.2049	R_1 = 0.0650, wR_2 = 0.1919
Final R indexes [all data]	R_1 = 0.0953, wR_2 = 0.2193	R_1 = 0.0365, wR_2 = 0.0882	R_1 = 0.0920, wR_2 = 0.2110	R_1 = 0.0739, wR_2 = 0.2010
Largest diff. peak/hole / e Å^{-3}	0.68/-0.61	0.19/-0.23	0.66/-0.52	0.31/-0.30

Supplementary Table 2. Structural parameters (θ, d_1 to d_3) of **3a** and **3b** from single crystal structures. θ is the torsion of C15C2C20C33 for **3a** and C1C3C37C21 for **3b**; d_1, d_2, and d_3 are the distances between C13 and C31, C8 and C26, and C9 and C27 for **3a** and C10 and C25 and C12 and C29 for **3b**.

	θ (degree)	d_1 (Å)	d_2 (Å)	d_3 (Å)
**3a**	11.4(2)	2.665(4)	3.618(4)	5.286(5)
**3b**	102.8(2)	3.313(3)	4.454(3)	-

Supplementary Table 3. Absolute calculation energies, enthalpies, and free energies

Geometry	$E_{(\text{elec-}\omega\text{B97X-D})}$[1]	$G_{(\text{corr-M11})}$[2]	$H_{(\text{corr-M11})}$[3]	IF[4]
**2a**	-730.19084363	0.196287	0.251336	-
**2b**	-808.82229672	0.246708	0.310234	-
**5**	-729.69604632	0.184202	0.237942	-
**6-ts**	-1459.90080234	0.407112	0.489574	-274.57
**7**	-1459.90961898	0.407612	0.49167	-
**8-ts**	-1459.89150650	0.406061	0.490097	-424.92
**9**	-1459.94866473	0.409678	0.492955	-
**10-ts**	-1459.89304701	0.406895	0.489805	-289.55
**11**	-1459.90937607	0.404599	0.491439	-
**12-ts**	-1459.88453571	0.407981	0.490057	-464.98
**13**	-1459.94742522	0.409688	0.492984	-
**14**	-808.32766852	0.236428	0.296797	-
**15-ts**	-1617.15800007	0.515743	0.608064	-306.59
**16**	-1617.17304592	0.515284	0.609308	-
**17-ts**	-1617.15484493	0.514248	0.607267	-484.52
**18**	-1617.21938620	0.517970	0.610086	-
**19-ts**	-1617.16261230	0.514181	0.607829	-271.54
**20**	-1617.17260023	0.516906	0.609563	-
**21-ts**	-1617.15336048	0.517156	0.610526	-427.25
**22**	-1617.21311845	0.518426	0.610475	-

[1] The electronic energy calculated by ωB97X-D/6-311+G(d,p) in methanol solvent. [2] The thermal correction to Gibbs free energy calculated by M11/6-31G(d) in methanol solvent. [3] The thermal correction to enthalpy calculated by M11/6-31G(d) in methanol solvent. [4] The M11

calculated imaginary frequencies for the transition states.

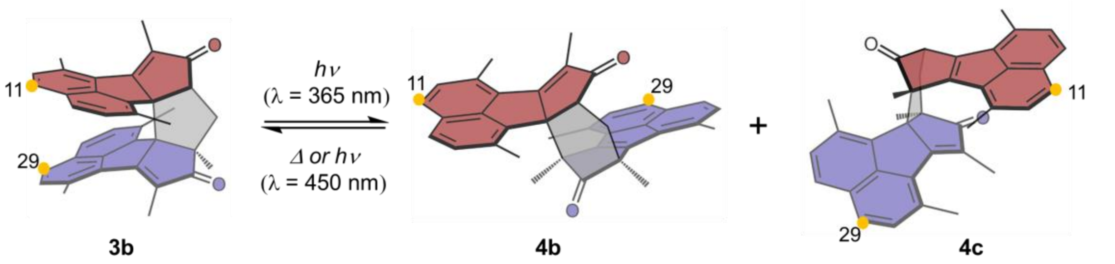

Supplementary Table 4. Comparison of properties of **3b**, **4b** and **4c**.

	**3b**	**4b**	**4c**
d_{C11C29}[1] (Å)	4.7	8.8	8.8
μ[1] (Debye)	6.2	2.7	2.8
LUMO[2] (eV)	–2.68	–2.82	
HOMO[2] (eV)	–5.51	–5.40	

[1] From optimized structures (ωB97X-D/6-31G(d,p) in the gas phase). [2] From cyclic voltammetries.

Supplementary Table 5. The fitting data of kinetics.

Equation	y = A1*exp(-kt)+y0			
Temperature (K)	A1	k	y0	R^2
**4a**				
303	2.87798	9.79730E-5	-0.01787	1.0000
308	2.75794	1.94163E-4	0.06959	1.0000
313	2.79101	3.77035E-4	0.05587	0.9999
318	2.93995	6.27952E-4	-0.04212	0.9999
323	2.85119	1.29438E-3	0.08329	0.9995
**4c**				
303	1.08044	9.04289E-5	-0.08007	1.0000
308	0.98715	1.91785E-4	0.01247	1.0000
313	1.02447	3.45079E-4	-0.01787	0.9981
318	1.00328	6.42035E-4	-0.00436	0.9999
323	1.01737	1.15571E-3	-0.01290	0.9991

Supplementary Methods

All commercially available chemicals were used without further purification unless otherwise noted. NMR spectra were recorded on Bruker AVANCE III-500 MHZ NMR, Bruker AVANCE III-600 MHZ NMR or AVANCE III 850 MHz NMR. ^{1}H NMR chemical shifts were referenced to $CDCl_3$ (7.26 ppm) or CD_2Cl_2 (5.32 ppm). ^{13}C NMR chemical shifts were referenced to $CDCl_3$ (77.0 ppm). High-resolution mass spectra (HRMS) were recorded on a Bruker En Apex Ultra 7.0T FT-MS mass spectrometer. Absorption spectra were recorded on a SHIMADA UV2550 UV-vis spectrometer. Circular dichroism (CD) spectra were recorded on a JASCO J-810 spectrometer. Cyclic voltammograms were recorded on a CHI660e electrochemical workstation. Single-crystal diffraction data were collected on a Rigaku SuperNova X-Ray single crystal diffractometer using Cu Kα (λ = 1.54184 Å) micro-focus X-ray sources at 150 K for **4b** and a XtaLAB Synergy, Dualflex, HyPix single crystal diffractometer using Cu Kα (λ =1.54184 Å) micro-focus sealed X-ray tube at 100 K for **3a**, **3b** and **4a**. High-performance liquid chromatography spectra were performed on SHIMADA LC-16A high-performance liquid chromatograph equipped with a Daicel CHIRALPAK IC or a Inertsil ODS-P column. The irradiation of the samples was carried out using four 1 W LEDs (λ = 365 nm, the four LEDs were arranged at a square pattern with a side length of about 1.4 cm, the radiation intensity at 6 cm is about 7.1 $mW\cdot cm^{-2}$), an 8 W UV lamp (λ = 254 nm) with a light filter or a 1 W LED (λ = 450 nm). The distances between the light sources and the samples were fixed at 6 cm. Compound **1b**[1] was synthesized as previously reported.

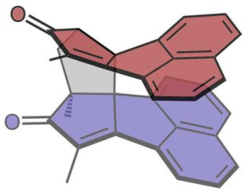

3a

Synthesis of 3a. A solution of KOH (862 mg, 15.4 mmol) in methanol (10 mL) was added dropwise to a mixture of **1a** (350 mg, 1.92 mmol), 3-pentanone (661 mg, 7.68 mmol) and methanol (10 mL) at room temperature. The mixture was stirred at reflux for 18 h until precipitation appeared. The solid was filtered, washed with water and dried. The crude product was further purified by column chromatography over neutral alumina (eluent: chloroform/hexane = 3/7) to give **3a** (168 mg, 38 %) as a yellow solid. ^{1}H NMR (500 MHz, CD_2Cl_2, 298 K, ppm) δ 7.41 – 7.32 (m, 3H), 7.30 – 7.16 (m, 7H), 7.16 – 7.11 (m, 1H), 7.07 (d, *J* = 6.9 Hz, 1H), 3.27 (d, *J* = 8.8 Hz, 1H), 3.04 (d, *J* = 13.8 Hz, 1H), 2.49 (dd, *J* = 13.8, 8.8 Hz, 1H), 1.97 (s, 3H), 1.92 (s, 3H), 1.22 (s, 3H). ^{13}C NMR (126 MHz, $CDCl_3$, 298 K, ppm) δ 211.73, 208.67, 173.26, 172.99, 142.41, 139.84, 139.77, 138.99, 134.85, 134.77, 131.01, 130.35, 130.29, 129.99, 127.40, 127.25, 127.02, 126.96, 126.70, 126.65, 123.39, 123.21, 123.12, 121.13, 120.11, 119.71, 73.56, 61.22, 57.58, 36.72, 23.65, 8.63, 8.52. HRMS (ESI, m/z): $[M+Na]^+$ calcd. for $C_{34}H_{24}O_2Na$, 487.1674; found 487.1678.

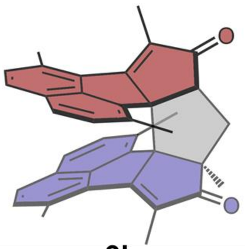

3b

Synthesis of 3b. A solution of KOH (747 mg, 13.3 mmol) in methanol (10 mL) was added dropwise to a mixture of **1b** (350 mg, 1.67 mmol), 3-pentanone (574 mg, 6.67 mmol) and methanol (10 mL) at room temperature. The mixture was then stirred at reflux for 18 h. The precipitation was filtered and purified by column chromatography over neutral alumina (eluent: chloroform/ hexane = 3/7) to give **3b** (277 mg, 64%) as a pale-yellow solid. ^{1}H NMR (500 MHz, CD_2Cl_2, 298 K, ppm) δ 7.42 (d, *J* = 8.3 Hz, 1H), 7.33 (d, *J* = 8.3 Hz, 1H), 7.27 (d, *J* = 8.2 Hz, 1H), 7.24 (d, *J* = 8.3 Hz, 1H), 7.18 (d, *J* = 8.3 Hz, 1H), 7.13 (d, *J* = 8.3 Hz, 1H), 6.78 (d, *J* = 8.2 Hz, 1H), 6.69 (d, *J* = 8.3 Hz, 1H), 3.57 (dd, *J* = 11.6, 5.5 Hz, 1H), 3.32 (dd, *J* = 15.0, 11.6 Hz, 1H), 2.46 (dd, *J* = 15.0, 5.5 Hz, 1H), 2.43 (s, 3H), 2.25 (s, 3H), 1.94 (s, 3H), 1.90 (s, 3H), 1.87 (s, 3H), 1.77 (s, 3H), 1.15 (s, 3H). ^{13}C NMR (101 MHz, $CDCl_3$, 298 K, ppm) δ 215.54, 211.61, 176.40, 174.35, 141.93, 140.77, 140.50, 138.36, 132.20, 132.04, 131.27, 130.68, 130.12, 130.07, 129.93, 129.53, 129.30, 129.13, 127.25, 126.85, 126.64, 125.65, 124.45, 123.83, 74.04, 63.21, 54.71, 34.49, 23.71, 22.84, 21.30, 20.77, 19.35, 11.06, 10.21. HRMS (ESI, m/z): $[M+H]^+$ calcd. for $C_{38}H_{33}O_2$, 521.2481; found 521.2478.

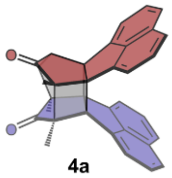

4a

Synthesis of 4a. A solution of **3a** (27.2 mg) in $CHCl_3$ (6 mL) was stirred and exposed to UV light for 80 min. The solvent was removed under vacumm to give the white solid **4a** (27 mg, 98%). ^{1}H NMR (500 MHz, $CDCl_3$, 298 K, ppm) δ 7.46 – 7.26 (m, 12H), 3.14 (dd, *J* = 6.0, 2.3 Hz, 1H), 2.95 – 2.84 (m, 2H), 1.67 (s, 3H), 1.63 (s, 3H), 1.08 (s, 3H). ^{13}C NMR (126 MHz, $CDCl_3$, 298 K, ppm) δ 221.38, 219.47, 143.41, 142.96, 142.26, 139.82, 137.92, 137.79, 132.02, 131.90, 127.99, 127.69, 127.62, 127.52, 124.33, 123.87, 123.86, 123.85, 122.23, 121.03, 120.94, 120.49, 71.50, 68.80, 63.21, 62.54, 60.85, 59.39, 59.20, 58.65, 52.81, 18.12, 11.55, 11.00. HRMS (ESI, m/z): $[M+Na]^+$ calcd. for $C_{34}H_{24}O_2Na$, 487.1674; found 487.1673.

Calculation details. All DFT calculations on mechanism were carried out with the Gaussian 09 series of programs (Gaussian 09, Revision D.01[2]). DFT method M11[3] with a standard 6-31G(d) basis set was used for geometry optimizations in the methanol solvent. Harmonic vibrational frequency calculations were performed for all of the stationary points to confirm them as a local minima or transition structures, and to derive the thermochemical corrections for the enthalpies and free energies. DFT method ωB97X-D with a standard 6-311G+(d,p) basis set was further used to calculate single-point energies. The solvent effects were considered by single point calculations

on the gas-phase stationary points with a SMD[4] solvation model (methanol).

Geometric optimizations of **3a**, **3b**, **4b**, and **4c** were also carried out at the long-range corrected functional ωB97X-D[5] with the 6-31G(d,p) basic set on Gaussian 09, Revision E.01[6] in the gas phase. Geometries of these molecules were confirmed with vibrational frequency calculations. The optimized geometries of **3a**, **3b**, **4b,** and **4c** were further used in TD-DFT calculations at the same level.

Single-crystal growth. The single crystals of **3a** and **3b** were obtained through the phase transfer of hexane into their dichloromethane solutions. The single crystal of **4a** was produced by the UV irradiation (λ = 365 nm, 3 h) of the single crystal of **3a**. The single crystal of **4b** was obtained from the phase transfer of methanol into the solution of the mixture of **4b** and **4c** in toluene at –19 °C.

Supplementary References

1. Butterfield, A. M., Gilomen, B.& Siegel, J. S. Kilogram-Scale Production of Corannulene. *Org. Process Res. Dev.* **16**, 664-676 (2012).

2. Gaussian 09, Revision D.01, M. J. Frisch, G. W. Trucks, H. B. Schlegel, G. E. Scuseria, M. A. Robb, J. R. Cheeseman, G. Scalmani, V. Barone, B. Mennucci, G. A. Petersson, H. Nakatsuji, M. Caricato, X. Li, H. P. Hratchian, A. F. Izmaylov, J. Bloino, G. Zheng, J. L. Sonnenberg, M. Hada, M. Ehara, K. Toyota, R. Fukuda, J. Hasegawa, M. Ishida, T. Nakajima, Y. Honda, O. Kitao, H. Nakai, T. Vreven, J. A. Montgomery, Jr., J. E. Peralta, F. Ogliaro, M. Bearpark, J. J. Heyd, E. Brothers, K. N. Kudin, V. N. Staroverov, T. Keith, R. Kobayashi, J. Normand, K. Raghavachari, A. Rendell, J. C. Burant, S. S. Iyengar, J. Tomasi, M. Cossi, N. Rega, J. M. Millam, M. Klene, J. E. Knox, J. B. Cross, V. Bakken, C. Adamo, J. Jaramillo, R. Gomperts, R. E. Stratmann, O. Yazyev, A. J. Austin, R. Cammi, C. Pomelli, J. W. Ochterski, R. L. Martin, K. Morokuma, V. G. Zakrzewski, G. A. Voth, P. Salvador, J. J. Dannenberg, S. Dapprich, A. D. Daniels, O. Farkas, J. B. Foresman, J. V. Ortiz, J. Cioslowski, and D. J. Fox, Gaussian, Inc., Wallingford CT, 2013.

3. Peverati, R.& Truhlar, D. G. M11-L: A Local Density Functional That Provides Improved Accuracy for Electronic Structure Calculations in Chemistry and Physics. *J. Phys. Chem. Lett.* **3**, 117-124 (2012).

4. Marenich, A. V., Cramer, C. J.& Truhlar, D. G. Universal Solvation Model Based on Solute Electron Density and on a Continuum Model of the Solvent Defined by the Bulk Dielectric Constant and Atomic Surface Tensions. *J. Phys. Chem. B* **113**, 6378-6396 (2009).

5. Chai, J.-D.& Head-Gordon, M. Long-range corrected hybrid density functionals with damped atom–atom dispersion corrections. *Phys. Chem. Chem. Phys.* **10**, 6615-6620 (2008).

6. Gaussian 09, Revision E.01, M. J. Frisch, G. W. Trucks, H. B. Schlegel, G. E. Scuseria, M. A. Robb, J. R. Cheeseman, G. Scalmani, V. Barone, B. Mennucci, G. A. Petersson, H. Nakatsuji, M. Caricato, X. Li, H. P. Hratchian, A. F. Izmaylov, J. Bloino, G. Zheng, J. L. Sonnenberg, M. Hada, M. Ehara, K. Toyota, R. Fukuda, J. Hasegawa, M. Ishida, T. Nakajima, Y. Honda, O. Kitao, H. Nakai, T. Vreven, J. A. Montgomery, Jr., J. E. Peralta, F. Ogliaro, M. Bearpark, J. J. Heyd, E. Brothers, K. N. Kudin, V. N. Staroverov, T. Keith, R. Kobayashi, J. Normand, K. Raghavachari, A. Rendell, J. C. Burant, S. S. Iyengar, J. Tomasi, M. Cossi, N. Rega, J. M. Millam, M. Klene, J. E. Knox, J. B. Cross, V. Bakken, C. Adamo, J. Jaramillo, R. Gomperts, R. E. Stratmann, O. Yazyev, A. J. Austin, R. Cammi, C. Pomelli, J. W. Ochterski, R. L. Martin, K. Morokuma, V. G. Zakrzewski, G. A. Voth, P. Salvador, J. J. Dannenberg, S. Dapprich, A. D. Daniels, O. Farkas, J. B. Foresman, J. V. Ortiz, J. Cioslowski, and D. J. Fox, Gaussian, Inc., Wallingford CT, 2013.
