# Supplementary material for: Unveiling how intramolecular stacking modes of covalently linked dimers dictate photoswitching properties
Source: Nat Commun. 2019 Dec 2;10:5480. doi: 10.1038/s41467-019-13428-3 (PMC6889182; doi:10.1038/s41467-019-13428-3)
Supplement: Supplementary file 2 — Description of Additional Supplementary Files [file 41467_2019_13428_MOESM2_ESM.pdf]

Description of Additional Supplementary Files

File Name: Supplementary Data 1
Description: The coordinates of optimized structures.

File Name: Supplementary Movie 1
Description: The photoisomerization from 3b to 4b.
